# Advancing Molecular Sieving via Å-Scale Pore Tuning in Bottom-Up Graphene Synthesis

**DOI:** 10.1021/acsnano.3c11885

**Published:** 2024-02-07

**Authors:** Cédric Van Goethem, Yueqing Shen, Heng-Yu Chi, Mounir Mensi, Kangning Zhao, Arian Nijmeijer, Paul-Emmanuel Just, Kumar Varoon Agrawal

**Affiliations:** † Laboratory for Advanced Separations (LAS), Institute of Chemical Sciences and Engineering (ISIC), Ecole Polytechnique Fédérale de Lausanne (EPFL), Rue de l’industrie 17, 1950 Sion, Switzerland; ‡ X-ray Diffraction and Surface Analytics Platform (XRD-SAP), Institute of Chemical Sciences and Engineering (ISIC), Ecole Polytechnique Fédérale de Lausanne (EPFL-Valais Wallis), Rue de l’industrie 17, 1950 Sion, Switzerland; § Shell Global Solutions International B.V., P.O. Box 38000, 1030 BN Amsterdam, The Netherlands; ∥ Inorganic Membranes, MESA+ Institute for Nanotechnology, University of Twente, P.O. Box 217, 7500 AE Enschede, The Netherlands

**Keywords:** graphene, membrane, gas separation, pore engineering, nickel

## Abstract

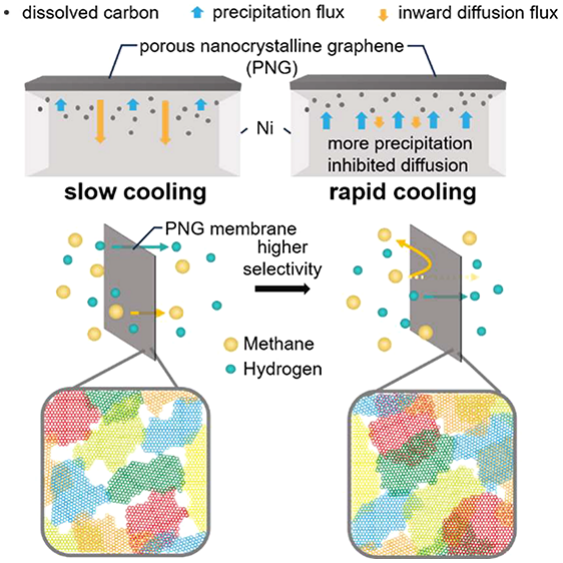

Porous graphene films are attractive as a gas separation membrane given that the selective layer can be just one atom thick, allowing high-flux separation. A favorable aspect of porous graphene is that the pore size, essentially gaps created by lattice defects, can be tuned. While this has been demonstrated for postsynthetic, top-down pore etching in graphene, it does not exist in the more scalable, bottom-up synthesis of porous graphene. Inspired by the mechanism of precipitation-based synthesis of porous graphene over catalytic nickel foil, we herein conceive an extremely simple way to tune the pore size. This is implemented by increasing the cooling rate by over 100-fold from −1 °C min^–1^ to over −5 °C s^–1^. Rapid cooling restricts carbon diffusion, resulting in a higher availability of dissolved carbon for precipitation, as evidenced by quantitative carbon-diffusion simulation, measurement of carbon concentration as a function of nickel depth, and imaging of the graphene nanostructure. The resulting enhanced grain (inter)growth reduces the effective pore size which leads to an increase of the H_2_/CH_4_ separation factor from 6.2 up to 53.3.

## Introduction

Membrane-based molecular separation is expected to play a crucial part in improving the energy-efficiency of several crucial processes in the transition to a sustainable society, e.g., hydrogen purification,^1,2^ carbon capture,^3^ sustainable resource recovery,^4,5^ flow batteries,^6^ remediation of emerging contaminants,^7^ etc. High-performance membranes that combine high permeance with high molecular specificity (with relative importance depending on the specific separation) are crucial to realize separation with a minimal energy penalty.^8,9^ Porous two-dimensional (2D)^10^ and other ultrathin materials (e.g., carbon nanomembranes^11,12^) have emerged as highly promising membrane selective layers, because mass transfer resistance scales inversely proportionally with the selective layer thickness. The 2D/ultrathin nature of the selective layer allows for the fabrication of thin and potentially highly permeable films. 2D materials used for membrane fabrication include metal–organic frameworks (MOFs),^13,14^ covalent–organic frameworks (COFs),^14,15^ zeolites,^16^ MXenes,^17^ graphene oxide,^18^ porous graphene,^19,20^ etc. The latter is especially interesting because it allows the selective layer to be as thin as the size of a single carbon atom. Nevertheless, a high density of molecular selective pores is a prerequisite for the potential of 2D membranes to fully materialize. Graphene has gained a lot of attention in the past two decades because of its interesting electronic, optical, mechanical, and chemical properties.^21^ While its atom-thick carbon lattice consisting of atoms arranged in a hexagonal lattice is impermeable to gases,^22,23^ incorporation of lattice vacancy defects as Å-scale pores renders porous graphene films highly promising for gas separation applications.^10^ Achieving graphene membranes with attractive gas separation performance generally relies on postsynthetic, top-down defect formation by etching the graphene lattice. A wide range of methods has been developed for this purpose including purely physical (e.g., ion-beam,^24^ electron-beam^25^) as well as oxidative chemical (e.g., O_3_^19,26,27^ or plasma etching^28^) routes. While the limitations in the control over pore size distribution and pore density in these strategies are continuously advanced,^10^ top-down pore-creation inherently increases the number of steps in material synthesis, complicating scale-up and increasing cost for large-scale graphene membrane production. In this context, bottom-up incorporation of a high density of gas-sieving pores concomitant to graphene synthesis could be highly advantageous.

Some progress has been made in increasing the density of intrinsic vacancy defects in the chemical vapor deposition (CVD) of graphene on Cu foil. This has been achieved by lowering the CVD temperature down to 800 °C^29,30^ or by using a larger hydrocarbon precursor such as benzene.^31^ Recently, bottom-up synthesis of porous graphene on a Ni foil by precipitation of a carbon precursor dissolved in Ni matrix was demonstrated.^32^ This approach is attractive as it effectively lowers the synthesis temperature to 500 °C, thereby, presenting an appealing opportunity to minimize the energy footprint of the process. The resulting film was termed as porous nanocrystalline graphene (PNG) because the graphene was composed of misoriented multilayered grains, which were only a few nanometers in size. So far, however, a strategy that allows control of pore size in graphene synthesized via the bottom-up route has not been reported. This is especially true for gas separation, where sub-Å resolution in molecular differentiation is needed.

Graphene growth on high-carbon-solubility catalysts such as Ni (but also Rh,^33^ Pt,^34^ etc.) differs significantly on various aspects from graphene crystallization on low carbon solubility catalysts such as Cu. Naturally all catalysts exhibit differences in carbon source adsorption and dehydrogenation,^35^ H_2_ adsorption and dissociation,^36^ as well as surface mobility of the active species. More importantly, catalysts with high carbon solubility can prompt a change in the growth mechanism from surface-mediated to precipitation-based growth. In this context, carbon will dissolve into the catalyst and diffuse away from the surface because of the carbon concentration gradient. Graphene can then segregate through two major segregation routes. (i) When carbon loading is low, there is an equilibrium segregation of the binary carbon–metal solution in which the graphene crystallizes during the heating and the isothermal phase of the synthesis.^37−40^ (ii) High carbon loading, on the other hand, triggers nonequilibrium segregation during cooling, referred to as precipitation.^41^ This is the main factor contributing to the commonly observed formation of multilayered graphene from high-carbon-solubility catalysts.^42^ High solubility has been also exploited to crystallize graphene via solid precursor transformation by placing the precursor on the opposite side of the catalytic metal foil.^40^ The high carbon solubility of nickel (∼0.4 atom % at 973 K^43^) in the presence of limited carbon precursor facilitates the formation of porous graphene such as PNG. Upon cooling, a sharp drop in carbon solubility initiates graphene precipitation with high nucleation density. The high nucleation density leads to nanometer-sized graphene grains, which are mostly multilayered but taper down to single layer graphene near the grain-edge. The grains are misoriented, which results in vacancy defects at the merger of two or more grains. When composed of 10 or more missing carbon atoms, these defects become attractive as gas permeable pores. These pores were shown to be capable of molecular sieving of gas molecules with attractive gas separation performance, especially when compared with other reports based on intrinsic defects in graphene.^32^ A brief comparison of top-down and bottom-up synthesis methods to incorporate pores in graphene is discussed in Table S6.

To improve control over the pore size distribution in PNG, an improved understanding of PNG crystallization is needed. Herein, we conceive a strategy to control grain growth by controlling the precipitation rate. This was achieved by drastically increasing the cooling rate by more than 100-fold which led to a sharp increase in gas pair selectivity. Quantitative carbon-diffusion simulation revealed a higher availability of dissolved carbon beneath the surface with an increased cooling rate, which could be experimentally verified through depth-resolved X-ray photoelectron spectroscopy (XPS). The higher extent of precipitation was confirmed by the visualization of a higher average number of graphene layers in rapidly cooled films using high-angle annular dark-field scanning transmission electron microscopy (HAADF-STEM).

## Results and Discussion

**Figure 1:**
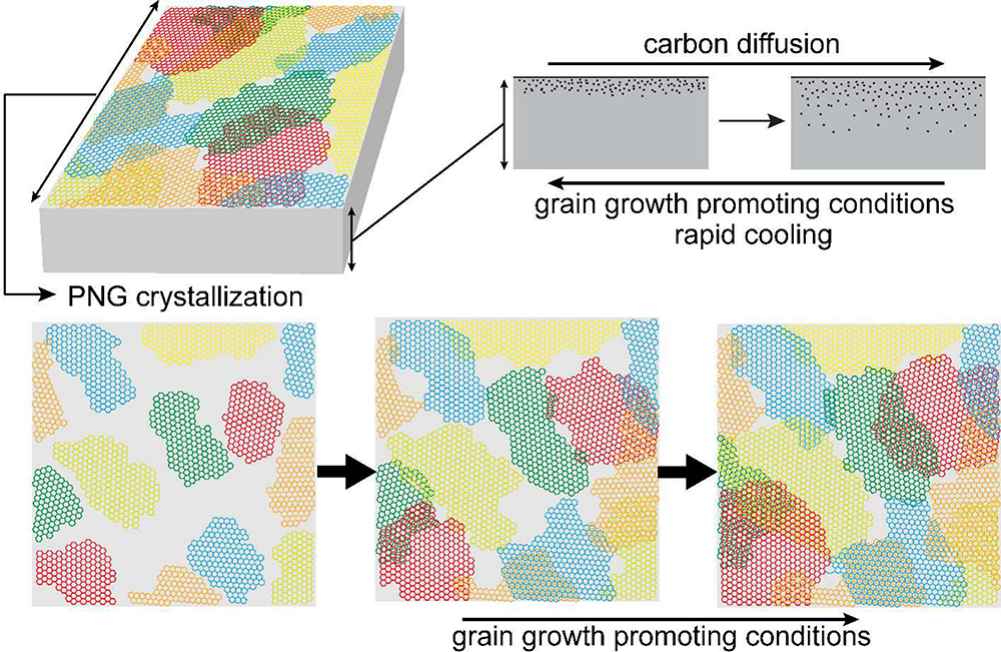
Schematic illustration of porous nanocrystalline graphene formation on a high-carbon-solubility substrate illustrating how tuning grain growth conditions can be used to control grain intergrowth and, hence, the resulting pore size.

The nucleation and growth mechanism resulting in a PNG film composed of “stitched nanosized graphene domains” does offer a path toward control over pore size. Pores are grain-boundary defects where a stronger (inter)growth would lead to smaller pores and vice versa. Therefore, promoting or restricting grain growth should allow control over the mean pore size. The easiest way to accomplish this is through control over carbon availability during crystallization and the degree of supersaturation that is realized. The seemingly most obvious route to achieve this would be through controlling the total amount of precursor, but most of the (additional) precursors in a pyrolysis process would probably simply gasify. We decided to follow an alternative, more powerful, route. The crystallization of PNG is realized during the cooling phase of the synthesis, in which the carbon solubility in the Ni matrix decreases drastically, triggering carbon supersaturation followed by precipitation. The heat of dissolution of carbon in nickel matrix is estimated to be near −0.42 eV, much lower than activation barrier for diffusion 1.74 eV.^43^ If cooling is carried out slowly, it provides a long time frame for carbon to diffuse into the bulk of the nickel, most of it too far to diffuse back to the surface at lower temperature when diffusivity plummets. Instead, cooling rapidly restricts the diffusion of carbon from the nickel surface. The resulting drop in solubility with a higher carbon concentration increases the degree of supersaturation, leading to stronger precipitation. This strategy, illustrated in Figure 1, is demonstrated in this work as a means of controlling pore size and, therefore, gas permeation properties.

**Figure 2:**
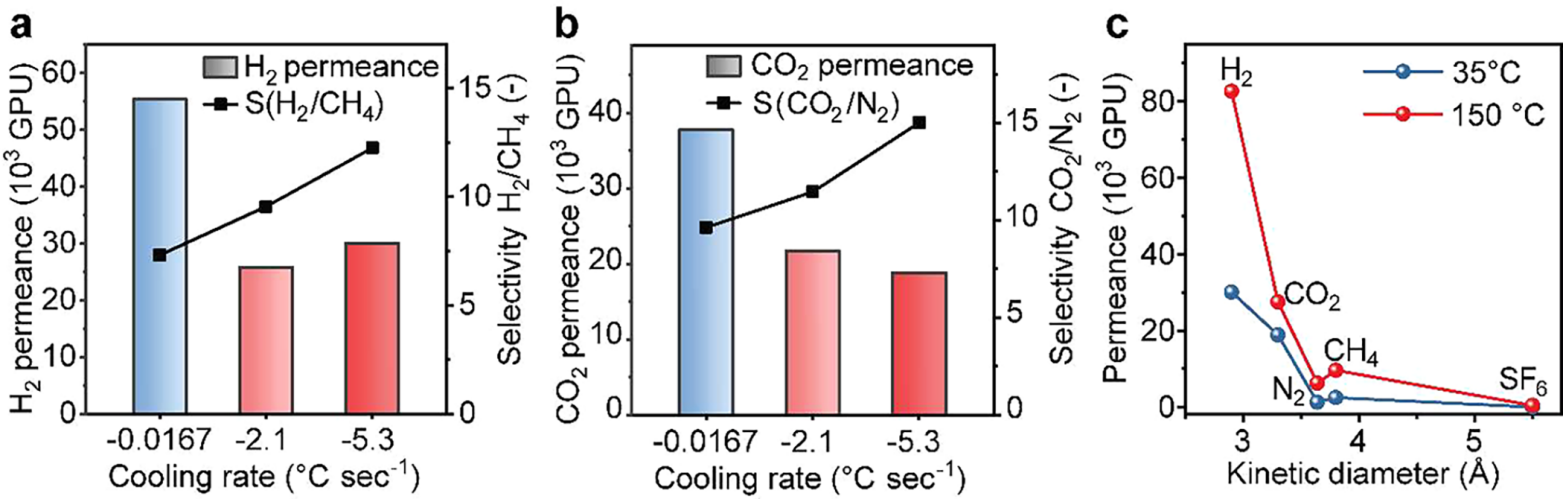
Effect of cooling rate on the permeation properties of non-reinforced PNG membranes for (a) H_2_/CH_4_ and (b) CO_2_/N_2_ separation. A detailed overview of the permeation properties as a function of gas kinetic diameter and temperature for the −5.3 °C s^–1^ non-reinforced PNG membrane is shown in panel c. The raw permeation data are compiled in the Supporting Information.

The initial investigation toward the effect of increased cooling on the properties of PNG was performed by simply opening the furnace and combining this with additional convective heat removal through application of compressed air. This resulted in a drastic increase in cooling rate from the reference −0.0167 °C s^–1^, referred to as “slow cooling”, to several °C s^–1^ (see the Supporting Information, Figure S2 and Table S1). The PNG films studied herein are prepared by the pyrolysis of a thin film of carbon precursor on 25 μm thick Ni foil. The precursor is a combination of turanose and a block copolymer [polystyrene-*block*-poly(4-vinylpyridine) or PS-*b*-P4VP]. The precursor pyrolysis results in a PNG film intimately connected to a pyrolyzed film of nanoporous carbon (NPC). The porosity in NPC is formed by templating the pyrolyzed carbon around the block copolymer. The NPC layer acts as a mechanical-reinforcing support film for PNG allowing one to achieve crack-free transfer of PNG by simply etching Ni and lifting PNG by the desired substrate. The PNG/NPC films were then transferred to a W foil hosting an array of 5 μm holes to probe gas transport properties. For this, the W foil was loaded in a membrane module, and the feed side was pressurized by the desired gas. The permeate (sweep) stream composition was followed by means of a mass spectrometer (see details in the SI). Films prepared using slow cooling yielded H_2_ permeance over 55 × 10^3^ GPU (1 GPU = 3.35 × 10^–10^ mol m^–2^ s^–1^ Pa^–1^) and H_2_/CH_4_ selectivity (*S*_H2/CH4_) of 7.3 (Figure 2a), consistent with the earlier report. Increasing the cooling rate by opening the furnace (−2.1 °C s^–1^) or by additional forced convection (−5.3 °C s^–1^) significantly enhanced the permeation properties. *S*_H_2_/CH_4__ increased from 7.3 to 9.5 and 12.2 for rapid cooling rates of −2.1 and −5.3 °C s^–1^, respectively, while the accompanying H_2_ permeance remained quite high (26 × 10^3^ and 30 × 10^3^ GPU, respectively). Similar trends were observed for CO_2_/N_2_ separation (Figure 2b) where *S*_CO_2_/N_2__ increased from 9.6 to 11.5 and 15.0, respectively.

Next, we studied the permeation properties of the rapidly cooled sample (−5.3 °C s^–1^) for several gases (H_2_, CO_2_, N_2_, CH_4_, and SF_6_) at different temperatures (Figure 2c). By plotting permeance as a function of the kinetic diameter of gas, we could clearly observe a sharp decline in permeance for the larger gas molecules. There was negligible permeance for the largest gas molecule that we could probe (SF_6_, kinetic diameter of 5.5 Å, H_2_/SF_6_ selectivity of 209 at 150 °C, Figure 2c). CH_4_ transported faster than N_2_ across PNG, consistent with a majority of the reports on transport through Å-scale graphene pores.^44−46^ This is attributed to the relatively stronger adsorption of CH_4_ on the graphitic lattice. We note that the NPC film does not contribute to the observed selectivity because it is not gas selective (H_2_/CH_4_ and CO_2_/N_2_ selectivities of 2.8 and 0.9, respectively, determined by Knudsen diffusivity through its channels which are several nanometers in size).^47^ The gas pair selectivities from PNG were much higher than the corresponding Knudsen selectivities, which indicates that the transport is determined by a strong confinement of the impingement rate of the molecules. To probe this further, we measured gas permeance at higher temperature, where permeance increased significantly, a characteristic of the activated transport.

**Figure 3:**
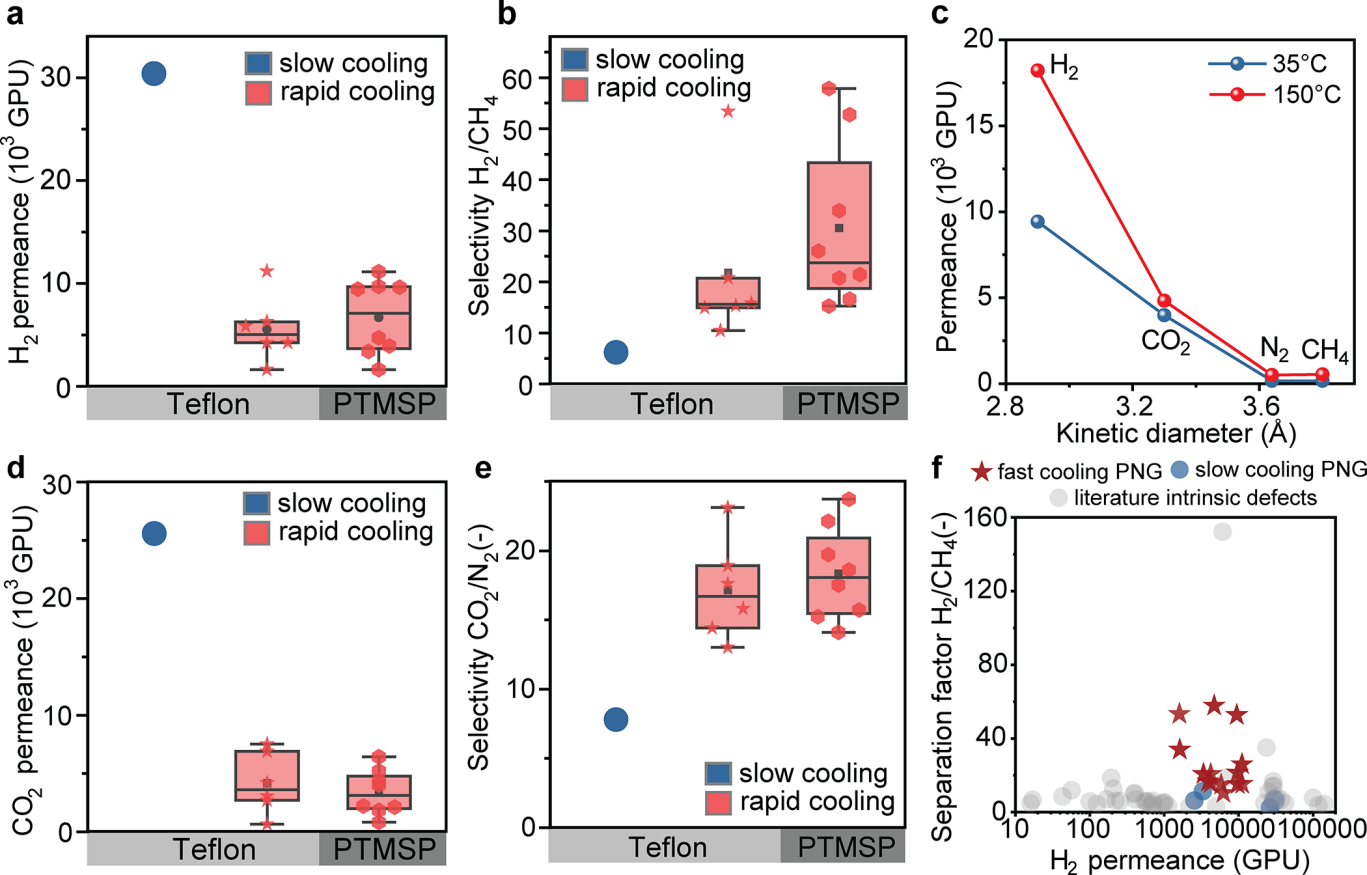
Effect of the cooling rate on the permeation properties of Teflon or PTMSP-reinforced PNG membranes. H_2_/CH_4_ (a, b) and CO_2_/N_2_ (d, e) separation performances of reinforced PNG membranes prepared using different cooling rates. A detailed overview of the permeation properties of a Teflon-reinforced −5 °C s^–1^ membrane at 35 and 150 °C is shown in panel c. (f) Comparison of the H_2_/CH_4_ performance of the herein obtained PNG membranes with previously reported PNG and other literature reports of SLG with solely intrinsic defects. Raw permeation data are compiled in the Supporting Information.

We did observe variance in the permeation properties of membranes prepared from the same batch of PNG. There is likely two sources of variance: first from the sample uniformity and second from the transfer of graphene to the macroporous support, which may create nanoscale defects in the film. Such nonselective defects have large permeance and can have a marked effect on selectivity. To reduce the stress generated during transfer, we reinforced the as-synthesized film with a thin film of a highly permeable polymer (Teflon AF 2400) resulting in a composite film structure of PNG/NPC/Teflon. This approach improved our success rate in membrane fabrication, defined as the percentage of successful transfer from 20% to above 80%. Here, successful transfer refers to the case when *S*_H_2_/CH_4__ of the composite membrane is greater than that from the standalone Teflon film (*S*_H_2_/CH_4__ = 5).^48^ We observed a significantly larger *S*_H_2_/CH_4__ (up to 53.3, Figure 3b) from the PNG/NPC/Teflon composite membrane using a rapid cooling rate of −5.3 °C s^–1^ compared to that (*S*_H_2_/CH_4__ = 6.2) obtained by slow cooling (−0.0167 °C s^–1^). We attempted to further increase the cooling rate to >−8 °C s^–1^, but this did not result in a further increase of selectivity. Presumably this is caused by the too fast cooling starting to restrain carbon supply toward the crystallization plane, thereby thwarting graphene precipitation (see the Supporting Information, Note S3). Another intriguing observation from the single-gas permeation test is that also the N_2_ permeance was diminished compared to that of CO_2_ (Figure 3c) which renders these films suitable for CO_2_/N_2_ separation. For CO_2_/N_2_ separation, poly(1-trimethylsilyl-1-propyne) or PTMSP was explored as an alternative reinforcing layer mainly because this polymer shows a higher intrinsic CO_2_/N_2_ selectivity (6–9^49−51^). Membranes were obtained with *S*_CO_2_/N_2__ up to 23.7 with an attractive CO_2_ permeance of 3980 GPU illustrating the potential of bottom-up synthesized porous graphene for carbon capture application. Notably, this study demonstrates the selectivity of CO_2_/N_2_ from porous graphene directly synthesized by the bottom-up approach. In fact, this separation performance is quite attractive for postcombustion capture and compares well with the porous graphene made by top-down postsynthetic etching of a graphene lattice.

The attractive H_2_/CH_4_ separation performance of PNG membranes prepared by rapid cooling is best illustrated when compared to literature data for the separation performance from intrinsic defects in graphene (Figure 3f). PNG membranes show an exciting performance based on their combination of very high H_2_/CH_4_ selectivity and excellent permeance, highlighting the advantages of the bottom-up approach toward nanoporous graphene.

To probe the mechanism behind improved selectivity from rapid cooling, we investigated PNG crystallization over a nickel substrate. For this, the dissolution and diffusion of carbon in nickel in relevant process conditions were modeled and complemented with experiments probing carbon distribution in the nickel foil and extent of graphene precipitation (discussed later).

**Figure 4:**
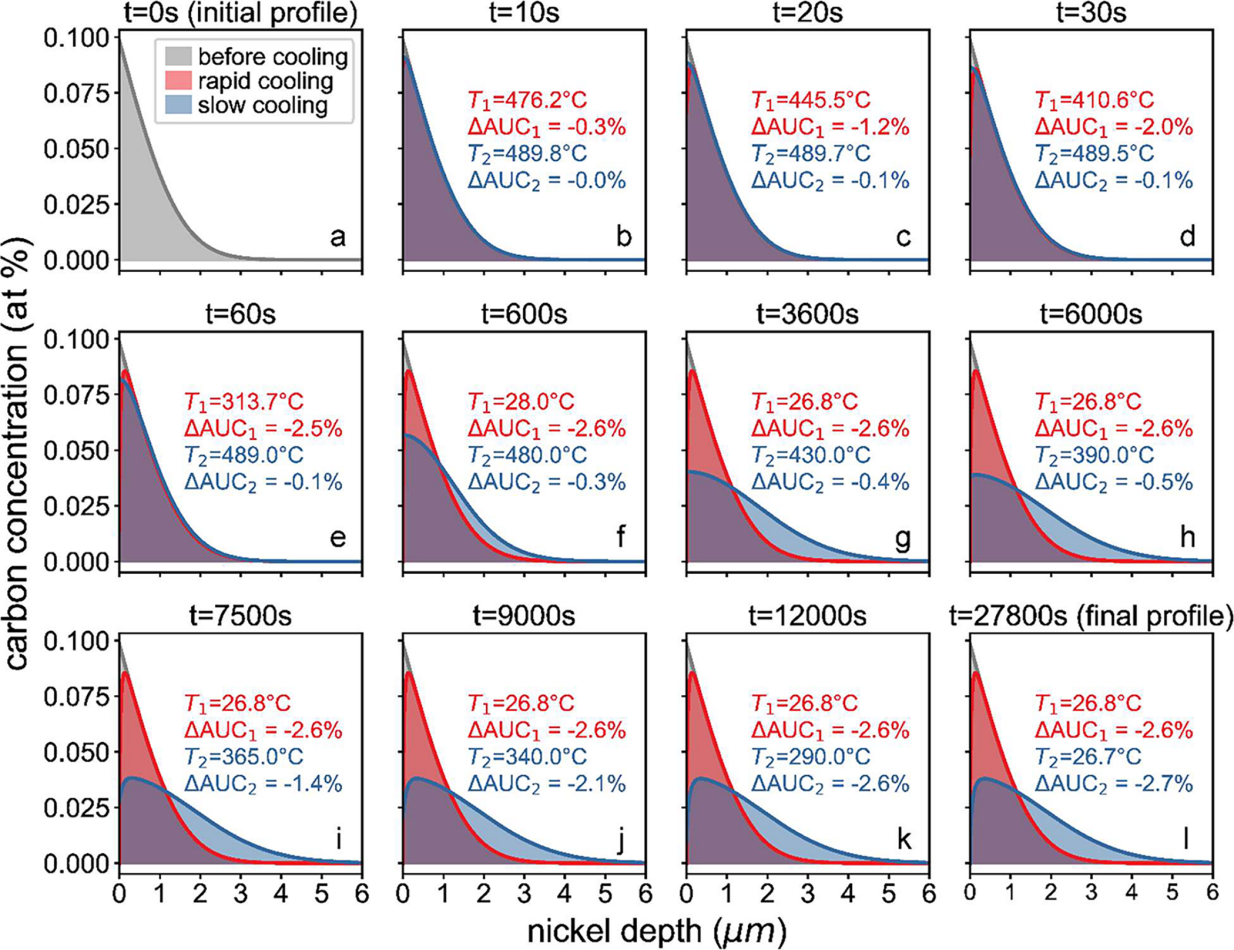
Simulated carbon concentration profile in different cooling stages where the blue curve represents the initial carbon profile after 10 min of annealing at 490 °C but before the cooling procedure, and the orange and green curves represent the carbon profile as the fast cooling and normal cooling proceed, respectively. The real-time temperature and change of curve under area (AUC) in the fast cooling case (red letters *T*_1_, ΔAUC_1_) and normal cooling case (blue, *T*_2_, ΔAUC_2_) are also shown.

The model simulated a nickel foil with a carbon source on one side, similar to the experiments. A two-stage CVD process was simulated including an initial carbon-loading “pyrolysis” and a subsequent cooling stage (details in Note S1). The diffusion and precipitation of carbon were modeled using Fick’s second law, solved by a finite difference method. The carbon concentration profile in the nickel substrate at various timeframes in the cooling process is shown in Figure 4 (full video is available in the Supporting Information) with the initial and final profile represented by panels a and l, respectively. A real-time change in the area under the carbon concentration curve (AUC) during cooling was computed as it provides a dynamic representation of the carbon content dissolved in the nickel. During the early stage of cooling, the carbon in the rapid cooling case quickly precipitated out of the nickel in the first 60 s (Figure 4a–e) as the ΔAUC_1_ changed from 0% to −2.5%, due to a rapid decrease in temperature and corresponding decrease in solubility. However, in the slow cooling case, given that the temperature during the first 3600 s was still high (430 °C), carbon mostly continued to diffuse deeper into the bulk nickel. No significant precipitation was observed at this point (−0.1%). This resulted in a lower carbon concentration at the surface, ∼50% of that in the rapid cooling case before precipitation (Figure 4e–h).

The temperature range during which precipitation occurs in the two cooling cases is drastically different. In the rapid cooling case, most precipitation took place during the cooling from 490 to 314 °C (Figure 4a–e) while, in the slow cooling case, most precipitation (2.1%) occurred during cooling from 390 to 290 °C (Figure 4i–l). It is worth noting that carbon solubility and diffusivity in nickel are highly sensitive to temperature, as the diffusivity at 390 °C (1.5 × 10^–17^ m^2^/s) is only 2% of the diffusivity at 490 °C (8 × 10^–16^ m^2^/s). Diffusivity plays a crucial role in the graphene growth, as demonstrated by a previous simulation study on graphene synthesized on nickel. It was found that, at a higher temperature, carbon atoms are sufficiently mobile with higher diffusivity to wander over the surface, facilitating the growth of a ring structure and resulting in less defective graphene.^52^ One could note that the model predicts a similar amount of overall precipitation for rapid and slow cooling (2.6% and 2.7% of the total amount of carbon initially present in the system, respectively). This is the result of the model allowing for precipitation even at low temperature, in combination with the much longer time for carbon diffusion in slow cooling. Indeed, on average, the temperature in the slow cooling case is 75 °C lower to reach similar levels of precipitation as for rapid cooling. This is not expected to reflect the experimental reality because (1) the model does not consider the influence of the degree of supersaturation on precipitation, and (2) below a certain temperature, the carbon mobility will become so low that in reality no precipitation would take place anymore.

**Figure 5:**
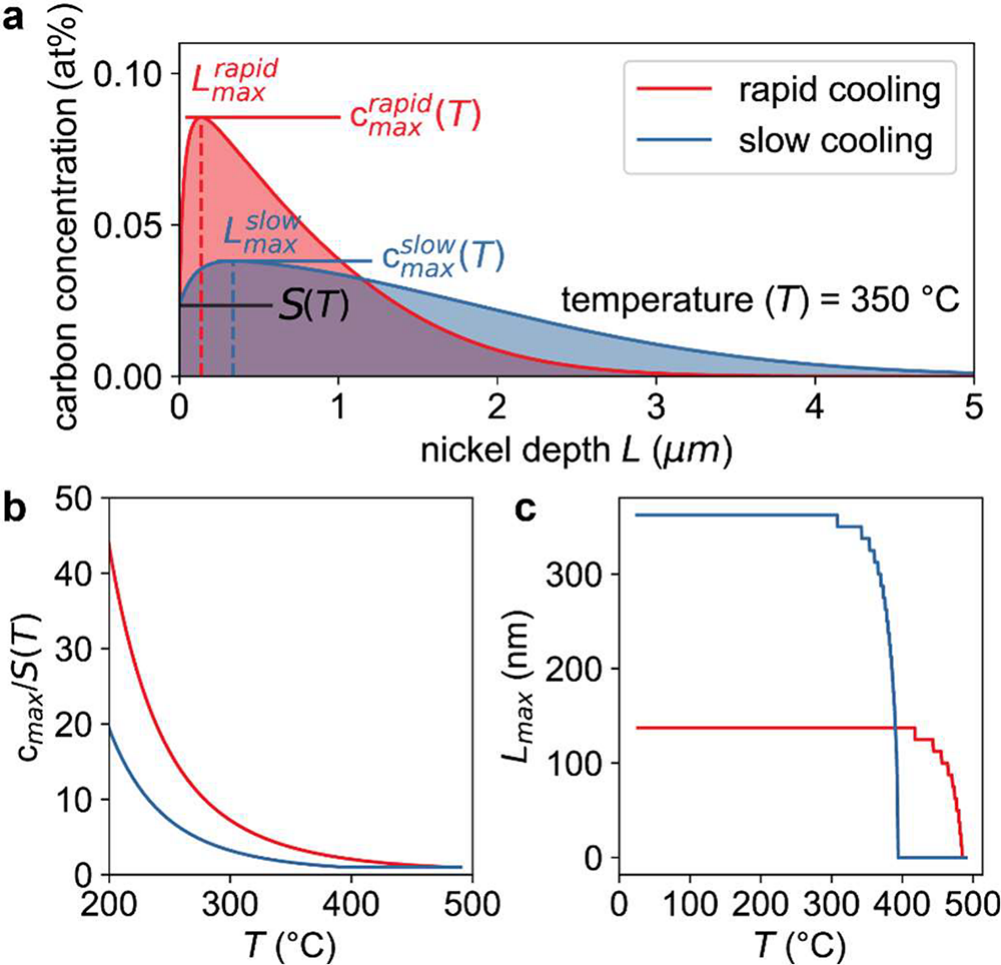
In-silico analysis of the carbon supersaturation degree. (a) Estimation of the degree of supersaturation as a ratio between the maximum carbon concentration inside the nickel foil over the carbon solubility for the two cooling cases at *T* of 350 °C. Evolution of the degree of supersaturation (b) and position of the *L*_max_(*T*) (c) as a function of temperature during the cooling process.

To probe the supersaturation at a given temperature, the ratio of the maximum carbon concentration inside the nickel foil (*c*_max_) to the carbon solubility was calculated (Figure 5a). From the evolution of the supersaturation degree during the cooling process (Figure 5b), one can observe that the degree of supersaturation for rapid cooling is approximately double that from the slow cooling. Plotting of the location of *c*_max_(*T*) [*L*_max_(*T*)] (Figure 5c) further substantiates that this supersaturation also stays closer to the surface of the Ni foil.

To validate the model-predicted trend in the carbon concentration, we prepared a film cross-section by a focused-ion beam and carried out annular dark-field scanning transmission electron microscopy (ADF-STEM). While the resulting image illustrated the PNG structure with its NPC reinforcing layer as well as the grain-structure of the underlying nickel foil (Figures S9 and S10), we faced challenges in resolving carbon concentration. This mainly originates from a lack of sensitivity as well as from contamination formed during preparation of the lamella by a focused-ion beam and during STEM itself.

**Figure 6:**
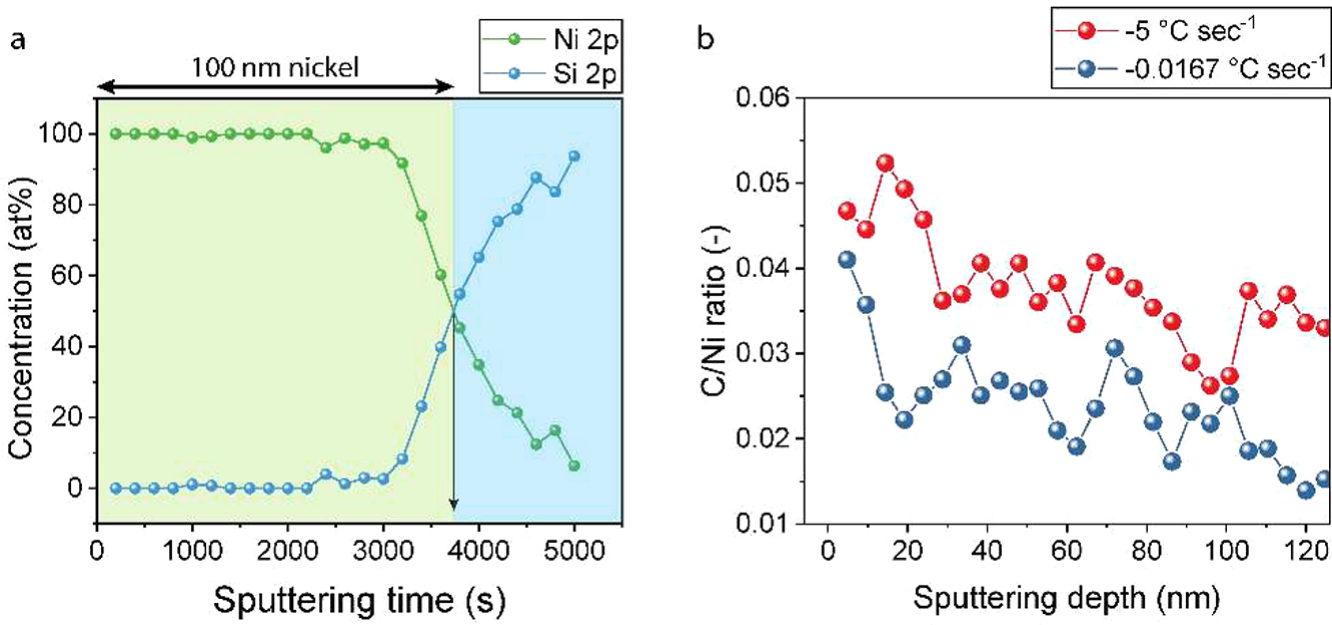
Ar^+^ sputtering-based depth-resolved XPS. (a) Ni 2p and Si 2p concentration as a function of 2 keV Ar^+^ sputtering time. The point where 50/50 concentration is reached is highlighted using a black arrow at 3737 s. (b) C 1s to Ni 2p ratio at increasing depth into the nickel foil of a PNG sample synthesized using −0.0167 and −5 °C s^–1^ cooling rates. The data is represented as a moving average of ±1 sputtering cycle to partially reduce the measurement noise.

As an alternative route to resolve carbon concentration as a function of depth, we pursued depth-resolved XPS, which has the advantage of improved sensitivity and cleaner “in situ” sample preparation. Nickel was etched in a controlled way using the Ar^+^ beam where the sputtering rate was calibrated using a reference 100 nm nickel on silicon wafer sample (Figure 6a). Subsequently, carbon-to-nickel (C/Ni) ratios in Ni foils were measured after PNG synthesis using slow and rapid cooling rates (Figure 6b). Indeed, a higher carbon concentration is found for the rapidly cooled sample over the complete measured range, confirming that indeed faster cooling leaves a higher amount of carbon near the surface as well as shows a higher carbon concentration at the nickel interface/crystallization plane. We note that the experimentally determined carbon concentration is much higher (±1.5–6 atom % over the first 120 nm) than expected based on the solubility at 500 °C (∼0.1 atom %). This discrepancy has been observed in the literature^53^ and is attributed to both intragrain precipitation in polycrystalline foils. We also consider that the role of surface contamination which manages to stay in the Ar^+^ bombarded zone could play a role here. Nevertheless, the trend from the in-depth XPS data is consistent with the model prediction.

As mentioned before, an as-synthesized PNG film also has a layer of ∼200 nm thick NPC film on top of PNG. As such, it is not possible to view the structure of PNG film. To understand porosity evolution as a function of cooling rate, we made a simple modification in the chemistry; instead of using the combination of turanose and PS-*b*-P4VP, we synthesized PNG films using exclusively PS-*b*-P4VP which resulted in a 2D PNG film without the 3D nanoporous NPC structure. While we expect that porosity of the resulting PNG film will be different than those made with turanose/PS-*b*-P4VP, trends in porosity by comparing samples produced at two different cooling rates can be made.

**Figure 7:**
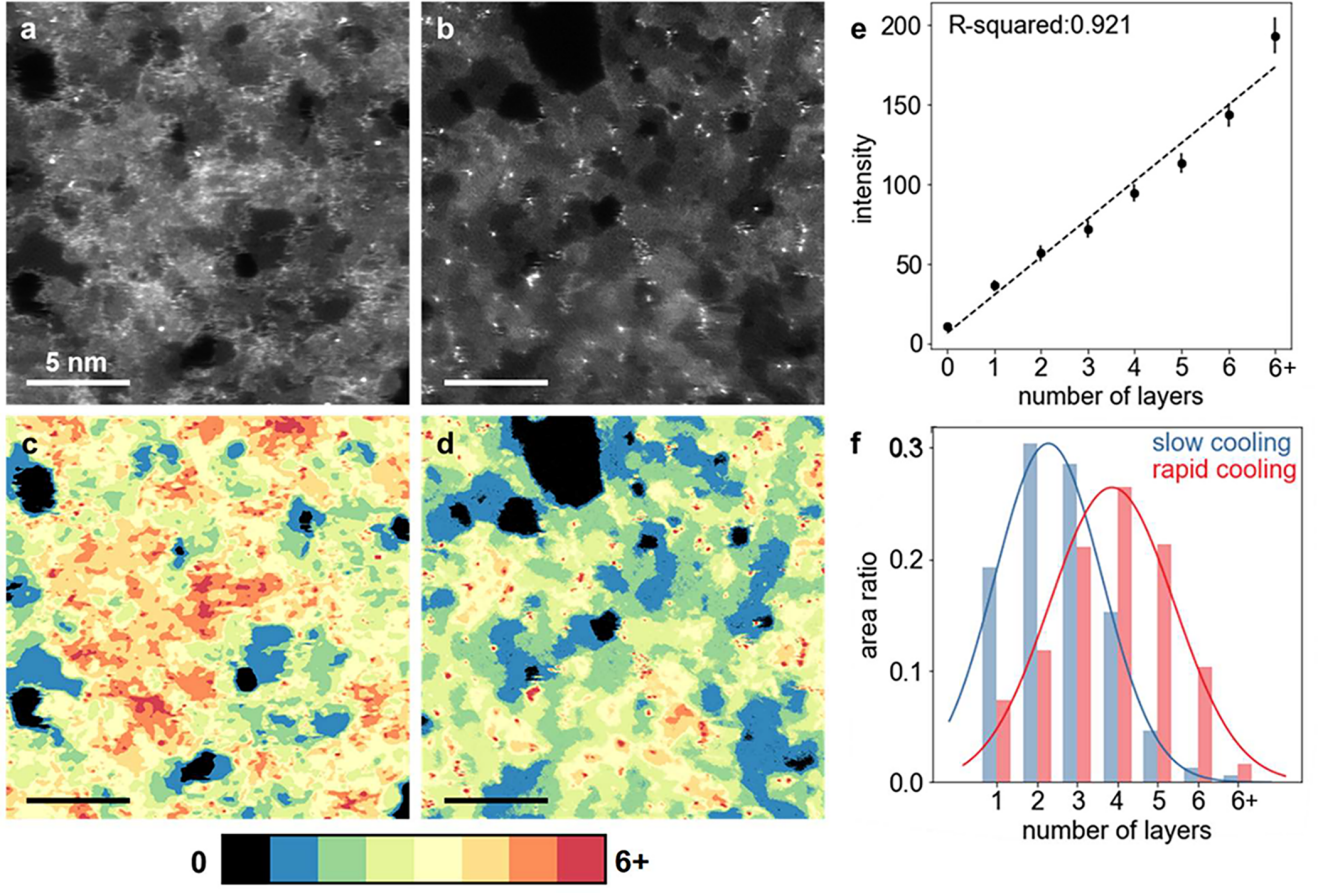
ADF-STEM images of a PNG film synthesized under different cooling conditions: (a) fast cooling and (b) normal cooling.^32^ A corresponding thickness map of the PNG-3 sample is displayed in panel c, where spectral colors indicate the number of graphene layers present. Black regions correspond to pores with no graphene layers, while blue to red regions represent 1 to 7 or more layers, respectively. (d) Graph indicating a direct relationship between the intensity and number of graphene layers in the PNG-3 film. The intensity for each layer was determined by calculating the average and standard deviation of 20 randomly selected pixels in each segmented layer group. (e) A histogram depicts the area ratio of each layer in normal and fast cooling conditions. The area ratio is calculated as the number of pixels for a specific layer divided by the total number of pixels in the image minus the number of pixels corresponding to pores. Scale bars in panels a–d are 5 nm.

ADF-STEM top-view images of the synthesized PNG samples in the absence of NPC film (Figure 7a,b) showed distinct microstructural features corresponding to two different cooling rates. Specifically, the PNG film synthesized using the −5 °C s^–1^ cooling condition was composed of more graphene layers than that synthesized using −0.0167 °C s^–1^ cooling. To quantify the distribution of graphene layers, the pixels of each ADF-STEM image were segmented into seven groups based on the number of graphene layers. The number of pixels in each group was determined, and the proportion of pixels in each group was calculated as the area ratio of graphene of different layers. The results are visualized by color mapping and shown in Figure 7c,d. To show the results more clearly, a histogram in Figure 7f displays the area ratio of each layer for both samples. This analysis confirms that, in the rapid cooling case, there are more multilayered areas, providing support for our hypothesis that, in this case, rapid carbon precipitation within a short time period helps to promote growth of graphene, resulting in a thicker PNG with more layers and shrinking pores.

Bottom-up synthesis of porous graphene is an intrinsically scalable technique as it cuts down an additional postsynthetic step on creating porosity in graphene. To further probe the uniformity and scalability of this synthesis approach, we prepared large coupons of PNG measuring 7.5 × 4 cm^2^ in dimension (Note S5). Membranes prepared by these larger coupons also yielded a promising CO_2_/N_2_ separation performance (Figure S8).

## Conclusions

In this work, we report the implementation of a simple yet effective strategy toward control over pore size and hence gas separation performance of bottom-up synthesized porous nanocrystalline graphene, which was established through altering the cooling rate after precursor pyrolysis. The cooling rate is an effective tool to favor or restrict carbon diffusion into the nickel substrate and hence the supply of carbon available for graphene crystallization through nonequilibrium segregation. Increased cooling rates were shown to promote growth of the nanometer-sized graphene domains with better intergrowth resulting in decreasing effective pore size. This resulted in a marked increase in the gas separation selectivity of PNG membranes where H_2_/CH_4_ selectivities in excess of 50 could be achieved. A combined in-silico and experimental investigation was used to gain quantitative insight into the underlying processes governing PNG growth and the final PNG structure. An increased amount of carbon precipitation was both predicted in-silico as well as observed experimentally through STEM-imaging which showed a higher average number of graphene layers. The simulation further suggested that the surface carbon concentration is higher when cooling faster, which could, aside from increased total carbon precipitation, also enhance nucleation density.

## Methods

### Materials

Nickel foils (99.9%, 25 μm thickness, electrodeposited) were purchased from Goodfellow Cambridge Ltd. Polystyrene-*block*-poly(4-vinylpyridine) (PS-*b*-P4VP) was sourced from Polymer Source Inc. (*M*_n_ PS = 11 800; *M*_n_ P4VP = 12 300; *M*_W_/*M*_n_ = 1.08). d-(+)-Turanose (98%) and iron(III) chloride (97%) were acquired from Sigma-Aldrich. Hydrochloric acid (HCl) (32%) and isopropanol (99%) were bought from Rectolab SA. Toluene was from Merck KGaA, Darmstadt. 4,5-Difluoro-2,2-bis(trifluoromethyl)-1,3-dioxole, polymer with tetrafluoroethylene (Teflon AF), and its corresponding perfluorinated solvent (Galden HT 110) were purchased from Chemours. Nitric acid (HNO_3_) and poly(1-trimethylsilyl-1-propyne) (PTMSP) were sourced from abcr. Dimethylformamide (DMF, 99.5%) was obtained from Roth.

### PNG Synthesis

PNG was synthesized through controlled pyrolysis of a precursor-coated nickel foil in a reducing atmosphere. Prior to precursor-coating, the nickel foil was annealed to remove contamination and to promote the (111) orientation. For this, the foils were sonicated for 15 min each in acetone and isopropanol, separately, to remove grease and other coarse contamination. The foils were then dried and heated to 1000 °C at 20 °C min^–1^ in a moderately oxidizing CO_2_ atmosphere, maintained between 600 and 700 Torr. At 1000 °C the atmosphere was changed to a reducing 9.1% H_2_ in Ar atmosphere before being heated further to 1100 °C at 10 °C min^–1^. The furnace was kept isothermally at 1100 °C for 2 h before cooling back to 1000 °C at −0.1 °C min^–1^ and finally to room temperature naturally. The foils were stored in a plastic vacuum desiccator until use.

The precursor solution for PNG synthesis was prepared by dissolving 0.15 g of PS-*b*-P4VP and 0.30 g of turanose in 2 g of DMF. After 1 h of sonication to facilitate dissolution, the solution was loaded into a Teflon-lined autoclave and heated to 180 °C for 3 h.

The annealed foils were coated with the precursor solution after mechanical polishing for 10 min to smooth the surface. The polishing was performed by using an MTI UNIPOL-1210 polishing apparatus using a diamond polishing paste (0.25 μm). The polished nickel foils were then sonicated again for 15 min in subsequent acetone and 2-propanol to remove residues from the polish before being taped to a glass slide to spin-coat the precursor. Spin-coating was performed for 1 min at 1000 rpm with 200 rpm/s acceleration. The films were stored in a plastic Petri dish for 1 h to allow the DMF to evaporate prior to loading into the pyrolysis furnace. A 7% H_2_ in Ar atmosphere was established at atmospheric pressure (100 sccm Ar/7.5 sccm H_2_), and the furnace was heated to 500 °C at 1 °C/min and kept isothermal for 10 min. In the so-called “slow cooling” process, the furnace is kept isothermal at 500 °C for a further 50 min before cooling to room temperature at −1 °C/min. Various faster cooling options were explored by cooling the furnace down after the 10 min isothermal phase at 500 °C by, e.g., opening the furnace, putting a fan in place, or applying an additional convective flow over the tube by means of compressed air.

### Membrane Preparation

PNG membranes were prepared by etching the nickel foil and subsequent rinsing and transfer to a suitable porous substrate. For etching, the PNG-on-nickel films were floated for 1 h on a 1 M aqueous FeCl_3_ solution that was filtered before use. The free-floating PNG films were then transferred subsequently to a 1 M HCl solution (1 h) and deionized water (1 h). The transfer was performed using a hydrophilized (plasma treated) piece of silicon wafer. Afterward, the PNG-films were scooped with a porous tungsten substrate for permeation testing. The 50 μm thick tungsten substrate features an array of 2500 laser-drilled 5 μm diameter holes over a 1 × 1 mm^2^ area.

In some cases (mentioned specifically in the main text), the PNG was further reinforced with a thin polymer layer to further reduce the formation of cracks during the transfer process. Both Teflon AF and PTMSP were used for this purpose. Teflon AF1600 (1 wt % in Galden HT 110) or PTMSP (3 wt % in toluene) coatings onto PNG were applied using a Laurell WS-650Mz-23NPPB spin-coater using, respectively, a 300 and 1000 rpm spin-coating speed for 1 min. The Teflon films were dried by heating for 3 h at 60 °C in a convection oven, whereas the PTMSP films were dried for at least 12 h in air and subsequently overnight in a vacuum oven.

### Characterization

#### Scanning Electron Microscopy (SEM)

SEM images were recorded by using an FEI Teneo microscope operated at 1 kV with a 25 pA current. The samples were mounted on a standard SEM stub and imaged without any additional coating.

#### (Scanning) Transmission Electron Microscopy (TEM/STEM)

PNG films were directly transferred to QUANTIFOIL R 1.2/1.3 grids and imaged using a double-corrected Titan Themis 60-300 (FEI, Thermo Fisher Scientific) equipped with a Wein-type monochromator. The transfer was carried out by the wet-transfer method without any reinforced layer. The specimens were heated in a heating holder from Gatan at 100 °C under vacuum (10^–3^ Pa) over 12 h prior to the imaging to reduce the atmospheric contamination. Aberration-corrected high-resolution transmission electron microscopy (AC-HRTEM) was carried out at 80 kV with a monochromated incident electron beam to reduce the chromatic aberration, and the negative spherical aberration (Cs) of ∼18 μm was applied to enhance the resolution of imaging. The electron dose rate was maintained at ∼1.6 × 10^4^ e^–^ Å^–2^ s^–1^ during imaging to reduce beam damage. AC scanning transmission electron microscopy (STEM) was performed at 80 kV to reduce the knock-on damage during acquisition. The probe convergence and STEM detector semiangles were, respectively, 20 and 49–200 mrad.

TEM lamella preparation: On top of PNG-2, either a Au layer was sputtered or an amorphous carbon layer was deposited (40 min electron beam deposition at 30 kV, followed by an ion beam deposition at 30 kV, 40 pA for the first 100 nm, and 150 pA for the following 900 nm). At 30 kV, a coarse milling and thinning procedure was conducted including 13 nA milling and 6.5 nA/3 nA/1.5 nA/0.7 nA thinning. A final low kV polishing step was performed under 5 kV 30 pA/80 pA for 10–20 s until the lamella was qualified for TEM imaging.

The electron energy loss spectroscopy (EELS) of PNG/Ni foil lamella was performed using a double-corrected Titan Themis 60-300 instrument (FEI, Thermo Fisher Scientific) at 300 kV. HAADF-STEM, bright-field TEM images, selected area electron diffraction (SAED), and energy dispersive X-ray spectroscopy (EDS) maps of PNG/Ni foil lamella were collected using a Talos F200S instrument (FEI, Thermo Fisher Scientific) at 200 kV.

#### X-ray Photoelectron Spectroscopy (XPS)

Chemical composition of the nickel foils after PNG synthesis was analyzed through depth-resolved XPS. A single piece of annealed nickel foil was washed consecutively with acetone and 2-propanol through sonication (15 min each) and then coated with the PNG precursor solution as described above. The polishing step was omitted in the XPS sample preparation to avoid the polishing process from influencing the structure/composition of the nickel foil. A single spin-coating step was done after which the coated sample was cut into two; one piece was subjected to PNG synthesis with a −1 °C min^–1^ cooling rate while another was cooled at −5 °C s^–1^. The foils were stored before and after synthesis in a vacuum desiccator. Immediately before XPS analysis, the PNG-layer was removed via exposure to a 15 wt % HNO_3_ aqueous solution. The samples were then rinsed with copious amounts of deionized water before being dried with compressed air and fixed onto the XPS sample holder. XPS analysis was performed using a Kratos Analytical Axis Supra instrument using the Al Kα X-ray line. Ni 2p and C 1s were measured with a pass energy and step-size of the analyzer of, respectively, 80 and 0.2 eV. Ar^+^ sputtering was performed at 2 keV over a 2.5 × 2.5 mm^2^ area in cycles of 180 s. The sputter rate was calibrated using a 100 nm Ni on a Si-wafer sputter-deposited sample.

### Gas Permeation

Membrane gas permeation properties were measured using an in-house built setup. The feed gas flow rate was kept at 30 mL min^–1^ with the feed pressure kept at 2 bar while the permeate was swept with an appropriate flow rate of argon (15–50 mL min^–1^) at atmospheric pressure (1 bar) and led to a Hiden HPR-20 R&D mass spectrometer for analysis.

## Supporting Information Available

Additional discussion of the numerical simulation of carbon diffusion (Note S1), CVD cooling rate (Note S2), cooling at −8.2 °C s^–1^ (Note S3), increasing the heating rate (Note S4), scaling up PNG synthesis (Note S5), a detailed overview of the membrane performance data, and a table (Table S6) comparing the top-down and bottom-up synthesis methods (PDF)
Video of the simulated carbon concentration profile as a function of time (MP4)

## References

[ref1] Villalobos L. F.; Hilke R.; Akhtar F. H.; Peinemann K. V. Fabrication of Polybenzimidazole/Palladium Nanoparticles Hollow Fiber Membranes for Hydrogen Purification. Adv. Energy Mater. 2018, 8 (3), 1701567 10.1002/aenm.201701567.

[ref2] Radmanesh F.; Tena A.; Sudhölter E. J. R.; Hempenius M. A.; Benes N. E. Nonaqueous Interfacial Polymerization-Derived Polyphosphazene Films for Sieving or Blocking Hydrogen Gas. ACS Appl. Polym. Mater. 2023, 5 (3), 1955–1964. 10.1021/acsapm.2c02022. 36935655 PMC10012169

[ref3] He G.; Huang S.; Villalobos L. F.; Vahdat M. T.; Guiver M. D.; Zhao J.; Lee W.; Mensi M.; Agrawal K. V. Synergistic CO _2_ Sieving from Polymer with Intrinsic Microporosity Masking Nanoporous Single Layer Graphene. Adv. Funct. Mater. 2020, 30 (39), 2003979 10.1002/adfm.202003979.

[ref4] DuChanois R. M.; Cooper N. J.; Lee B.; Patel S. K.; Mazurowski L.; Graedel T. E.; Elimelech M. Prospects of Metal Recovery from Wastewater and Brine. Nat. Water 2023, 1 (1), 37–46. 10.1038/s44221-022-00006-z.

[ref5] Wang R. Performance Metrics for Nanofiltration-Based Selective Separation for Resource Extraction and Recovery. Nat. Water 2023 13 2022, 1 (3), 291–300. 10.1038/s44221-023-00037-0.

[ref6] Ye C.; Wang A.; Breakwell C.; Tan R.; Grazia Bezzu C.; Hunter-Sellars E.; Williams D. R.; Brandon N. P.; Klusener P. A. A.; Kucernak A. R.; Jelfs K. E.; McKeown N. B.; Song Q. Development of Efficient Aqueous Organic Redox Flow Batteries Using Ion-Sieving Sulfonated Polymer Membranes. Nat. Commun. 2022, 13 (1), 1–13. 10.1038/s41467-022-30943-y. 35676263 PMC9177609

[ref7] Ilyas S.; Abtahi S. M.; Akkilic N.; Roesink H. D. W.; de Vos W. M. Weak Polyelectrolyte Multilayers as Tunable Separation Layers for Micro-Pollutant Removal by Hollow Fiber Nanofiltration Membranes. J. Membr. Sci. 2017, 537, 220–228. 10.1016/j.memsci.2017.05.027.

[ref8] Marchetti P.; Peeva L.; Livingston A. The Selectivity Challenge in Organic Solvent Nanofiltration: Membrane and Process Solutions. Annu. Rev. Chem. Biomol. Eng. 2017, 8, 473–497. 10.1146/annurev-chembioeng-060816-101325. 28511021

[ref9] Shi B.; Marchetti P.; Peshev D.; Zhang S.; Livingston A. G. Will Ultra-High Permeance Membranes Lead to Ultra-Efficient Processes? Challenges for Molecular Separations in Liquid Systems. J. Membr. Sci. 2017, 525, 35–47. 10.1016/j.memsci.2016.10.014.

[ref10] Villalobos L. F.; Babu D. J.; Hsu K. J.; Van Goethem C.; Agrawal K. V. Gas Separation Membranes with Atom-Thick Nanopores: The Potential of Nanoporous Single-Layer Graphene. Accounts Mater. Res. 2022, 3 (10), 1073–1087. 10.1021/accountsmr.2c00143. PMC9623591 36338295

[ref11] Ai M.; Shishatskiy S.; Wind J.; Zhang X.; Nottbohm C. T.; Mellech N.; Winter A.; Vieker H.; Qiu J.; Dietz K. J.; Gölzhäuser A.; Beyer A. Carbon Nanomembranes (CNMs) Supported by Polymer: Mechanics and Gas Permeation. Adv. Mater. 2014, 26 (21), 3421–3426. 10.1002/adma.201304536. 24535992

[ref12] Qi Y.; Yang Y.; Westphal M.; Ennen I.; Cremer J.; Anselmetti D.; Hütten A.; Gölzhäuser A.; Dementyev P. Pyrene-Derived Carbon Nanomembranes Selectively Pass Metal Ions in Water. Adv. Mater. Interfaces 2022, 9 (27), 2201385 10.1002/admi.202201385.

[ref13] Liu Q.; Miao Y.; Villalobos L. F.; Li S.; Babu D. J.; Chi H.-Y.; Vahdat M. T.; Hao J.; Song S.; Han Y.; Tsapatsis M.; Agrawal K. V. Unit-Cell-Thick Zeolitic Imidazolate Framework Films for Membrane Application. Nat. Mater. 2023, 22 (11), 1387–1393. 10.1038/s41563-023-01669-z. 37735526 PMC10627807

[ref14] Knebel A.; Caro J. Metal-Organic Frameworks and Covalent Organic Frameworks as Disruptive Membrane Materials for Energy-Efficient Gas Separation. Nature Nanotechnology 2022, 17, 911–923. 10.1038/s41565-022-01168-3. 35995854

[ref15] Fan H.; Mundstock A.; Feldhoff A.; Knebel A.; Gu J.; Meng H.; Caro J. Covalent Organic Framework-Covalent Organic Framework Bilayer Membranes for Highly Selective Gas Separation. J. Am. Chem. Soc. 2018, 140 (32), 10094–10098. 10.1021/jacs.8b05136. 30021065

[ref16] Agrawal K. V.; Zhang X.; Elyassi B.; Brewer D. D.; Gettel M.; Kumar S.; Lee J. A.; Maheshwari S.; Mittal A.; Sung C.-Y.; Cococcioni M.; Francis L. F.; McCormick A. V.; Mkhoyan K. A.; Tsapatsis M. Dispersible Exfoliated Zeolite Nanosheets and Their Application as a Selective Membrane. Science 2011, 334 (6052), 72–75. 10.1126/science.1208891. 21980106

[ref17] Shao D.-D.; Zhang Q.; Wang L.; Wang Z.-Y.; Jing Y.-X.; Cao X.-L.; Zhang F.; Sun S.-P. Enhancing Interfacial Adhesion of MXene Nanofiltration Membranes via Pillaring Carbon Nanotubes for Pressure and Solvent Stable Molecular Sieving. J. Membr. Sci. 2021, 623, 119033 10.1016/j.memsci.2020.119033.

[ref18] Wang S.; Mahalingam D.; Sutisna B.; Nunes S. P. 2D-Dual-Spacing Channel Membranes for High Performance Organic Solvent Nanofiltration. J. Mater. Chem. A 2019, 7 (19), 11673–11682. 10.1039/C8TA10872B.

[ref19] Huang S.; Dakhchoune M.; Luo W.; Oveisi E.; He G.; Rezaei M.; Zhao J.; Alexander D. T. L.; Züttel A.; Strano M. S.; Agrawal K. V. Single-Layer Graphene Membranes by Crack-Free Transfer for Gas Mixture Separation. Nat. Commun. 2018, 9 (1), 2632 10.1038/s41467-018-04904-3. 29980683 PMC6035196

[ref20] Kidambi P. R.; Mariappan D. D.; Dee N. T.; Vyatskikh A.; Zhang S.; Karnik R.; Hart A. J. A Scalable Route to Nanoporous Large-Area Atomically Thin Graphene Membranes by Roll-to-Roll Chemical Vapor Deposition and Polymer Support Casting. ACS Appl. Mater. Interfaces 2018, 10 (12), 10369–10378. 10.1021/acsami.8b00846. 29553242

[ref21] Geim A. K. Graphene: Status and Prospects. Science 2009, 324 (5934), 1530–1534. 10.1126/science.1158877. 19541989

[ref22] Bunch J. S.; Verbridge S. S.; Alden J. S.; Zande A. M. v. d.; Parpia J. M.; Craighead H. G.; McEuen P. L. Impermeable Atomic Membranes from Graphene Sheets. Nano Lett. 2008, 8 (8), 2458–2462. 10.1021/nl801457b. 18630972

[ref23] Sun P. Z.; Yang Q.; Kuang W. J.; Stebunov Y. V.; Xiong W. Q.; Yu J.; Nair R. R.; Katsnelson M. I.; Yuan S. J.; Grigorieva I. V.; Lozada-Hidalgo M.; Wang F. C.; Geim A. K. Limits on Gas Impermeability of Graphene. Nature 2020, 579 (7798), 229–232. 10.1038/s41586-020-2070-x. 32161387

[ref24] Celebi K.; Buchheim J.; Wyss R. M.; Droudian A.; Gasser P.; Shorubalko I.; Kye J.-I.; Lee C.; Park H. G. Ultimate Permeation Across Atomically Thin Porous Graphene. Science 2014, 344 (6181), 289–292. 10.1126/science.1249097. 24744372

[ref25] Garaj S.; Hubbard W.; Reina A.; Kong J.; Branton D.; Golovchenko J. A. Graphene as a Subnanometre Trans-Electrode Membrane. Nature 2010, 467 (7312), 190–193. 10.1038/nature09379. 20720538 PMC2956266

[ref26] Hsu K. J.; Villalobos L. F.; Huang S.; Chi H. Y.; Dakhchoune M.; Lee W. C.; He G.; Mensi M.; Agrawal K. V. Multipulsed Millisecond Ozone Gasification for Predictable Tuning of Nucleation and Nucleation-Decoupled Nanopore Expansion in Graphene for Carbon Capture. ACS Nano 2021, 15 (8), 13230–13239. 10.1021/acsnano.1c02927. 34319081 PMC8388115

[ref27] Huang S.; Villalobos L. F.; Li S.; Vahdat M. T.; Chi H. Y.; Hsu K. J.; Bondaz L.; Boureau V.; Marzari N.; Agrawal K. V. In Situ Nucleation-Decoupled and Site-Specific Incorporation of Å-Scale Pores in Graphene Via Epoxidation. Adv. Mater. 2022, 34 (51), 2206627 10.1002/adma.202206627. 36271513

[ref28] Boutilier M. S. H.; Jang D.; Idrobo J. C.; Kidambi P. R.; Hadjiconstantinou N. G.; Karnik R. Molecular Sieving Across Centimeter-Scale Single-Layer Nanoporous Graphene Membranes. ACS Nano 2017, 11 (6), 5726–5736. 10.1021/acsnano.7b01231. 28609103

[ref29] Yuan Z.; He G.; Faucher S.; Kuehne M.; Li S. X.; Blankschtein D.; Strano M. S. Direct Chemical Vapor Deposition Synthesis of Porous Single-Layer Graphene Membranes with High Gas Permeances and Selectivities. Adv. Mater. 2021, 33 (44), 2104308 10.1002/adma.202104308. 34510595

[ref30] Kidambi P. R.; Nguyen G. D.; Zhang S.; Chen Q.; Kong J.; Warner J.; Li A. P.; Karnik R. Facile Fabrication of Large-Area Atomically Thin Membranes by Direct Synthesis of Graphene with Nanoscale Porosity. Adv. Mater. 2018, 30 (49), 1804977 10.1002/adma.201804977. 30368941

[ref31] Khan M. H.; Moradi M.; Dakhchoune M.; Rezaei M.; Huang S.; Zhao J.; Agrawal K. V. Hydrogen Sieving from Intrinsic Defects of Benzene-Derived Single-Layer Graphene. Carbon N. Y. 2019, 153, 458–466. 10.1016/j.carbon.2019.07.045.

[ref32] Villalobos L. F.; Goethem C. V.; Hsu K. J.; Li S.; Moradi M.; Zhao K.; Dakhchoune M.; Huang S.; Shen Y.; Oveisi E.; Boureau V.; Agrawal K. V. Bottom-up Synthesis of Graphene Films Hosting Atom-Thick Molecular-Sieving Apertures. Proc. Natl. Acad. Sci. U. S. A 2021, 118 (37), e2022201118 10.1073/pnas.2022201118. 34493654 PMC8449330

[ref33] Liu M.; Zhang Y.; Chen Y.; Gao Y.; Gao T.; Ma D.; Ji Q.; Zhang Y.; Li C.; Liu Z. Thinning Segregated Graphene Layers on High Carbon Solubility Substrates of Rhodium Foils by Tuning the Quenching Process. ACS Nano 2012, 6 (12), 10581–10589. 10.1021/nn3047154. 23157621

[ref34] Sun J.; Nam Y.; Lindvall N.; Cole M. T.; Kenneth K. B.; Park Y. W.; Yurgens A. Growth Mechanism of Graphene on Platinum: Surface Catalysis and Carbon Segregation. Appl. Phys. Lett. 2014, 104 (15), 42 10.1063/1.4871978.

[ref35] An W.; Zeng X. C.; Turner C. H. First-Principles Study of Methane Dehydrogenation on a Bimetallic Cu/Ni(111) Surface. J. Chem. Phys. 2009, 131 (17), 174702 10.1063/1.3254383. 19895030

[ref36] Losurdo M.; Giangregorio M. M.; Capezzuto P.; Bruno G. Graphene CVD Growth on Copper and Nickel: Role of Hydrogen in Kinetics and Structure. Phys. Chem. Chem. Phys. 2011, 13 (46), 20836–20843. 10.1039/c1cp22347j. 22006173

[ref37] Shelton J. C.; Patil H. R.; Blakely J. M. Equilibrium Segregation of Carbon to a Nickel (111) Surface: A Surface Phase Transition. Surf. Sci. 1974, 43 (2), 493–520. 10.1016/0039-6028(74)90272-6.

[ref38] Isett L. C.; Blakely J. M. Segregation Isosteres for Carbon at the (100) Surface of Nickel. Surf. Sci. 1976, 58 (2), 397–414. 10.1016/0039-6028(76)90478-7.

[ref39] Eizenberg M.; Blakely J. M. Carbon Monolayer Phase Condensation on Ni(111). Surf. Sci. 1979, 82 (1), 228–236. 10.1016/0039-6028(79)90330-3.

[ref40] Weatherup R. S.; Baehtz C.; Dlubak B.; Bayer B. C.; Kidambi P. R.; Blume R.; Schloegl R.; Hofmann S. Introducing Carbon Diffusion Barriers for Uniform, High-Quality Graphene Growth from Solid Sources. Nano Lett. 2013, 13 (10), 4624–4631. 10.1021/nl401601x. 24024736 PMC3813970

[ref41] Li X.; Cai W.; Colombo L.; Ruoff R. S. Evolution of Graphene Growth on Ni and Cu by Carbon Isotope Labeling. Nano Lett. 2009, 9 (12), 4268–4272. 10.1021/nl902515k. 19711970

[ref42] Yu Q.; Lian J.; Siriponglert S.; Li H.; Chen Y. P.; Pei S. S. Graphene Segregated on Ni Surfaces and Transferred to Insulators. Appl. Phys. Lett. 2008, 93 (11), 113103 10.1063/1.2982585.

[ref43] Lander J. J.; Kern H. E.; Beach A. L. Solubility and Diffusion Coefficient of Carbon in Nickel: Reaction Rates of Nickel-Carbon Alloys with Barium Oxide. J. Appl. Phys. 1952, 23 (12), 1305–1309. 10.1063/1.1702064.

[ref44] Huang S.; Li S.; Villalobos L. F.; Dakhchoune M.; Micari M.; Babu D. J.; Vahdat M. T.; Mensi M.; Oveisi E.; Agrawal K. V. Millisecond Lattice Gasification for High-Density CO 2 - and O 2 -Sieving Nanopores in Single-Layer Graphene. Sci. Adv. 2021, 7 (9), eabf0116 10.1126/sciadv.abf0116. 33627433 PMC7904253

[ref45] Huang S.; Li S.; Hsu K. J.; Villalobos L. F.; Agrawal K. V. Systematic Design of Millisecond Gasification Reactor for the Incorporation of Gas-Sieving Nanopores in Single-Layer Graphene. J. Membr. Sci. 2021, 637, 119628 10.1016/j.memsci.2021.119628.

[ref46] Bondaz L.; Ronghe A.; Li S.; Čerņevičs K.; Hao J.; Yazyev O. V. Selective Photonic Gasification of Strained Oxygen Clusters on Graphene for Tuning Pore Size in Å-Regime. JACS Au 2023, 3 (10), 2844 10.1021/jacsau.3c00395. 37885574 PMC10598578

[ref47] Dakhchoune M.; Duan X.; Villalobos L. F.; Hsu K.-J.; Zhao J.; Micari M.; Agrawal K. V. Rapid Gas Transport from Block-Copolymer Templated Nanoporous Carbon Films. Ind. Eng. Chem. Res. 2021, 60 (44), 16100–16108. 10.1021/acs.iecr.1c03039.

[ref48] Pinnau I.; Toy L. G. Gas and Vapor Transport Properties of Amorphous Perfluorinated Copolymer Membranes Based on 2,2-Bistrifluoromethyl-4,5-Difluoro-1,3-Dioxole/Tetrafluoroethylene. J. Membr. Sci. 1996, 109 (1), 125–133. 10.1016/0376-7388(95)00193-X.

[ref49] Takada K.; Matsuya H.; Masuda T.; Higashimura T. Gas Permeability of Polyacetylenes Carrying Substituents. J. Appl. Polym. Sci. 1985, 30 (4), 1605–1616. 10.1002/app.1985.070300426.

[ref50] Ichiraku Y.; Stern S. A.; Nakagawa T. An Investigation of the High Gas Permeability of Poly (1-Trimethylsilyl-1-Propyne). J. Membr. Sci. 1987, 34 (1), 5–18. 10.1016/S0376-7388(00)80017-4.

[ref51] Srinivasan R.; Auvil S. R.; Burban P. M. Elucidating the Mechanism(s) of Gas Transport in Poly[1-(Trimethylsilyl)-1-Propyne] (PTMSP) Membranes. J. Membr. Sci. 1994, 86 (1–2), 67–86. 10.1016/0376-7388(93)E0128-7.

[ref52] Neyts E. C.; Van Duin A. C. T.; Bogaerts A. Formation of Single Layer Graphene on Nickel under Far-from-Equilibrium High Flux Conditions. Nanoscale 2013, 5 (16), 7250–7255. 10.1039/c3nr00153a. 23695014

[ref53] Bleu Y.; Barnier V.; Christien F.; Bourquard F.; Loir A. S.; Garrelie F.; Donnet C. Dynamics of Carbon Diffusion and Segregation through Nickel Catalyst, Investigated by in Situ XPS, during Growth of Nitrogen Doped Graphene. Carbon N. Y. 2019, 155, 410–420. 10.1016/j.carbon.2019.08.084.

