# Supplementary material for: Advancing Molecular Sieving via Å-Scale Pore Tuning in Bottom-Up Graphene Synthesis
Source: ACS Nano. 2024 Feb 7;18(7):5730–40. doi: 10.1021/acsnano.3c11885 (PMC10883125; doi:10.1021/acsnano.3c11885)
Supplement: Supplementary file 1 — nn3c11885_si_001.pdf [file nn3c11885_si_001.pdf]

Supporting Information

Advancing molecular sieving via Å-scale pore tuning in bottom-up graphene synthesis

*Cédric Van Goethem‡[a], Yueqing Shen‡[a], Heng-Yu Chi[a], Mounir Mensi[b], Kangning Zhao[a], Arian Nijmeijer[c,d], Paul-Emmanuel Just[c] and Kumar Varoon Agrawal[a]**

[a] Laboratory for Advanced Separations (LAS), Institute of Chemical Sciences and Engineering (ISIC), Ecole Polytechnique Fédérale de Lausanne (EPFL), Rue de l'industrie 17, 1950 Sion, Switzerland.

[b] X-ray diffraction and Surface Analytics Platform (XRD-SAP), Institute of Chemical Sciences and Engineering (ISIC), Ecole Polytechnique Fédérale de Lausanne (EPFL-Valais Wallis), Rue de l'industrie 17, 1950 Sion, Switzerland

[c] Shell Global Solutions International B.V., P.O. Box 38000, 1030 BN Amsterdam, the Netherlands

[d] Inorganic Membranes, MESA+ Institute for Nanotechnology, University of Twente, P.O. Box 217, AE Enschede 7500, the Netherlands

1. Supplementary notes 2
1.1. Supplementary Note S1: Numerical simulation of carbon diffusion 2
1.2. Supplementary Note S2: CVD cooling rate 6
1.3. Supplementary Note S3: cooling at -8.2 °C sec^{-1} 7
1.4. Supplementary Note S4: Increasing the heating rate 8
1.5. Supplementary Note S5: Scaling up PNG synthesis 10
2. Detailed overview of membrane data 13
3. Comparison of various synthesis procedure (Table S6) 17

1. Supplementary notes

1.1. Supplementary Note S1: Numerical simulation of carbon diffusion

The model is established on a nickel foil of thickness $l = 25\ \mu m$, similar to the experiment, with a carbon source on one side of the foil (Fig. S1a). To mimic the synthesis of PNG, a two-stage CVD process was simulated including (i) an initial stage: a t_1-minute annealing at 490°C when carbon atoms from the carbon source on one side dissolve and diffuse into Ni bulk, followed by (ii) a cooling stage: a t_2-minute rapid/slow cooling step when the dissolved carbon atoms precipitate to form the PNG. To focus on the effect of diffusion and precipitation on carbon concentration, the second Fick's law (equation **1**) was solely used:

$$\frac{\partial c}{\partial t} = D(T)\frac{\partial c^2}{\partial^2 x} \tag{1}$$

where $c = c(x, t)$ is the carbon concentration at depth x and $D(t)$ is the carbon diffusion coefficient at kelvin temperature T at the given time t. The concentration as a function of thickness, for a given time t, varies as c_1, c_2, c_3, …., c_n (Figure S1a).

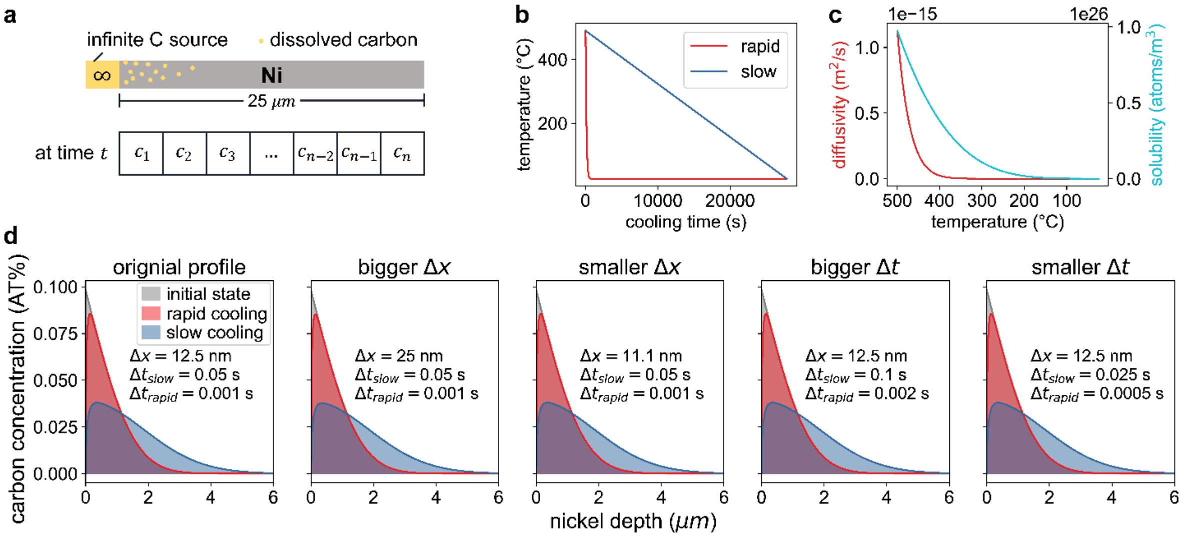

Figure S1. (a) A schematic of the numerical model. An example of discretization at time t is shown below; (b) Cooling rate in fast cooling and normal cooling, measured through a thermocouple in CVD; (c)Diffusion and solubility profile as a function of temperature; (d) Validation of simulation results by altering the discretization size in both time and space.

We make several assumptions in setting up the model which are listed below:

- The diffusion of carbon atoms through grain boundaries of nickel is neglected because nickel grains are much larger (several micron) than grain of PNG (2-3 nm).
- There is no mass exchange on the backside of nickel given that there is no solid precursor on the back side. This is validated by the carbon diffusion profile where the maximum carbon penetration depth is only 4 μm in comparison of the thickness of the foil which is 25 μm.
- The temperature change across the body of nickel is expected to be uniform given the high thermal conductivity of nickel.
- There is only inward diffusion of carbon during the annealing (J_{in}) while only outward precipitation (J_{out}) during the cooling, which are constrained in the boundary conditions.

The solubility and diffusivity of C in Ni can be expressed as:

$$S = S_0 exp(H/kT) \tag{3}$$

$$D = D_0 exp(-E_D/kT) \tag{4}$$

where $S_0 = 5.33 \times 10^{22} atoms/cm^3$ is an entropic prefactor associated with the density of sites where solute atoms can locate; $H = -0.42\ eV$ is the heat of dissolution; $D_0 = 2.48\ cm^2/s$; $E_D = 1.74\ eV$; k the Boltzmann's constant and T the kelvin temperature.[1]

D and S are a function of t since the temperature T also varies with time, and is programmed by CVD process:

$$T = f_{CVD}(t) \tag{2}$$

The time-temperature function of annealing, slow cooling, and fast cooling was based on time-resolved temperature measurement in the real PNG synthesis (Fig. S1b).

The boundary conditions for two different states are listed as follows:

(a) initial stage: carbon dissolution

$$c(0,t) = S(T)\ (0 \le t \le t_1)$$

$$c(x,0) = 0\ (0 < x \le L)$$

$$\frac{\partial c(x,t)}{\partial x}|_{x=L} = 0\ (0 < t \le t_1)$$

(b) cooling stage: carbon precipitation

$$c(x,0) = c(x,t_1), (0 < x \le l)$$

$$c(0,t) = S(T)\ (t_1 \le t \le t_1 + t_2)$$

$$J_{out,x=0} \propto \frac{\partial c(x,t)}{\partial x}|_{x=0} \ge 0\ (t_1 \le t \le t_1 + t_2)$$

$$\frac{\partial c(x,t)}{\partial x}|_{x=L} = 0\ (t_1 < t \le t_1 + t_2)$$

A finite difference method was employed to solve the above problems by discretizing both the nickel thickness L into n finite volume elements (FVEs) of thickness Δx and the reaction time into m FVEs of time interval Δt:

$$x_i = i\Delta x\ \ (i = 1,2, \dots, n)$$

$$t_j = j\Delta t\ \ (j = 1,2, \dots, m)$$

and the equation becomes:

$$c_i^j = c(x_i, t_j)$$

$$\frac{c_i^{j+1} - c_i^j}{\Delta t} = D(t_j)\frac{c_{i+1}^j - 2c_i^j + c_{i-1}^j}{\Delta x^2}$$

The boundary condition becomes:

$$\frac{c_1 - c_0}{\Delta x} \geq 0$$

The concentration at time t_{j+1} can be computed by the following equation and the results were achieved after $m \times n$ iteration.

$$c_i^{j+1} = D(t_j)\frac{\Delta t}{\Delta x^2}\left(c_{i+1}^j - 2c_i^j + c_{i-1}^j\right) + c_i^j$$

All numerical simulation experiments were conducted through a Python code. The 25 μm nickel was discretized into 2000 FVEs in space ($\Delta x = 12.5\ nm$) for both fast and slow cooling. Discretizating size of FVE in time was carefully selected based on different cooling rates. For normal cooling (from 490 °C to 25 °C, cooling rate 1°C min^{-1}), the time consumption of the whole cooling procedure (27900 seconds) was discretized into 558000 small FVEs ($\Delta t_{norm} = 0.05s$) in time while in fast cooling, the time (700 seconds) was discretized into 700000 small FVEs ($\Delta t_{fast} = 0.001s$) as the cooling rate is much faster(~150 °C min^{-1}). The results were validated by the fact that altering the size of spatial discretization ($\Delta x = 11.1\ nm, 25\ nm$) and time discretization ($\Delta t_{norm} = 0.025, 0.1s, ; \Delta t_{fast} = 0.0005, 0.002s$) didn't change the result carbon profile (Fig S1d).

1.2. Supplementary Note S2: CVD cooling rate

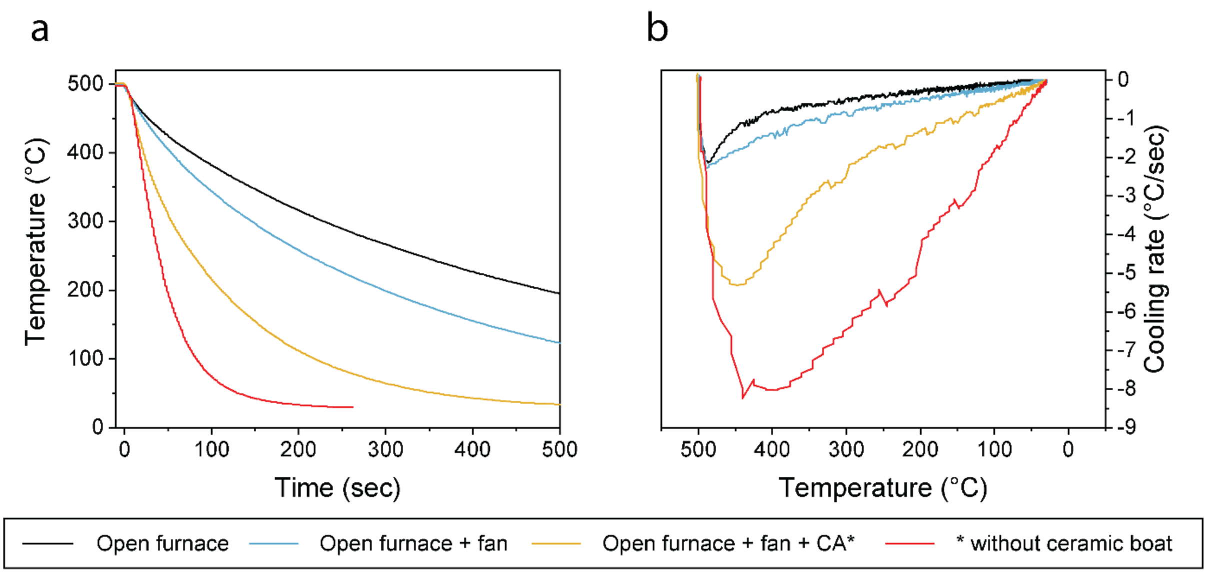

Figure S2. Temperature measurements of the different cooling strategies used in this work represented temperature evolution with time (a) and cooling rate at different temperatures (b). The cooling rate plots are represented as a 9-point moving average to flatten noise in the measurements.

Table S1. Numeric data of the different cooling strategies tested.

n°	Strategy	Max dT/dt (°C sec^{-1})	t to 400°C (sec)
1	-1 °C min^{-1} #	- 0.0167	100
2	Open furnace	-2.1	79
3	Open furnace + fan	-2.3	54
4	Open furnace + fan + CA*	-5.3	22
5	4 + without ceramic boat	-8.2	18

# values for the -1°C min^{-1} case are calculated as the cooling rate in this case is furnace program controlled.

* CA = compressed air

1.3. Supplementary Note S3: cooling at -8.2 °C sec^{-1}

Using forced convection cooling through compressed air, the main resistance towards cooling comes from the heat stored inside the ceramic boat (used to hold the precursor-coated nickel foil). Therefore, we attempted to further increase the cooling rate via the elimination of the ceramic boat. While indeed this resulted in a significant further increase in cooling rate from -5.3 to -8.2 °C sec^{-1} (see Note S2), permeation properties of these films were inferior to the samples prepared at -5.3 °C sec^{-1} and resemble closely to the PNG membranes using slow cooling (Figure S3). While this could possibly be ascribed to less even cooling without the balancing effect of the heat in the boat negatively impacting PNG quality, literature also suggests that too fast cooling could thwart graphene crystallization in non-equilibrium segregation because the fast cooling rate can limit carbon supply towards to the crystallization plane as a consequence of the rapidly dropping diffusivity eliminating carbon mobility. This has been reported to lead to incomplete graphene layer growth.[2] Additional cooling rate-related effects include the fact that high cooling rate can be accompanied by thermal gradients, which would promote carbon diffusion towards the bulk[3], as well as a reduced ability to crystalize into graphene by the rapidly dropping mobility of the carbon atoms.[4]

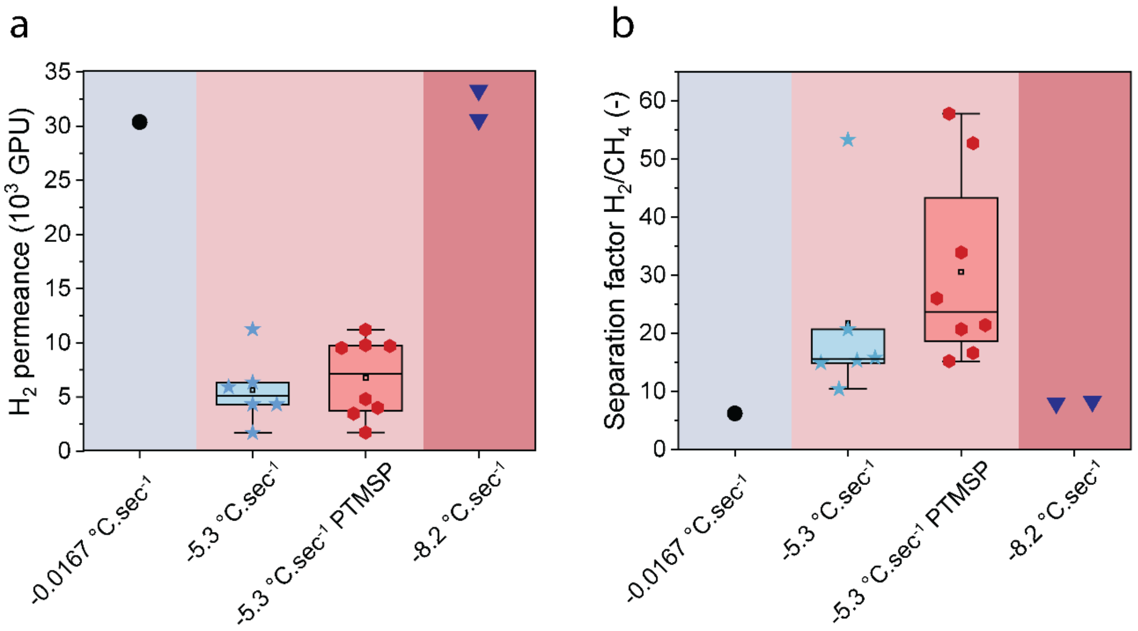

Figure S3. Effect of cooling rate on permeation properties of Teflon or PTMSP-reinforced PNG membranes for H_2/CH_4 separation.

1.4. Supplementary Note S4: Increasing the heating rate

Aside from the increased cooling rate, we also explored modifying the synthesis conditions by increasing the heating rate (from 1 to 2 and 5 °C min^{-1}) in the PNG synthesis while keeping a rapid cooling rate (-5.3 °C sec^{-1}). Higher heating rate appeared promising because:

(i) Increasing heating rate reduces the time to reach high temperature. Therefore, it reduces the ability for carbon to diffuse away from the interface.

(ii) The maximum pyrolysis rate when heating at 5 °C min^{-1} occurs around 389 °C, a higher temperature compared to 1 °C min^{-1} heating where pyrolysis maximizes before reaching 346 °C (Figure S4). Therefore, it increases the carbon uptake of the nickel foil because of an increased solubility of nickel at higher temperature.

Nevertheless, increasing the heating rate to 2 or 5 °C min^{-1} resulted in to a slightly decreased selectivity compared to our control case (heating at 1 °C min^{-1} and cooling at -5.3 °C sec^{-1}, Figure S5), suggesting the average pore size increased against expectation. A possible hypothesis for this is slow growth of graphene from a reduced carbon uptake of the nickel foil. The latter may happen because of a shift in pyrolysis products towards lighter (and thus more volatile) components which do not adsorb on Ni as strongly as heavier hydrocarbon product.

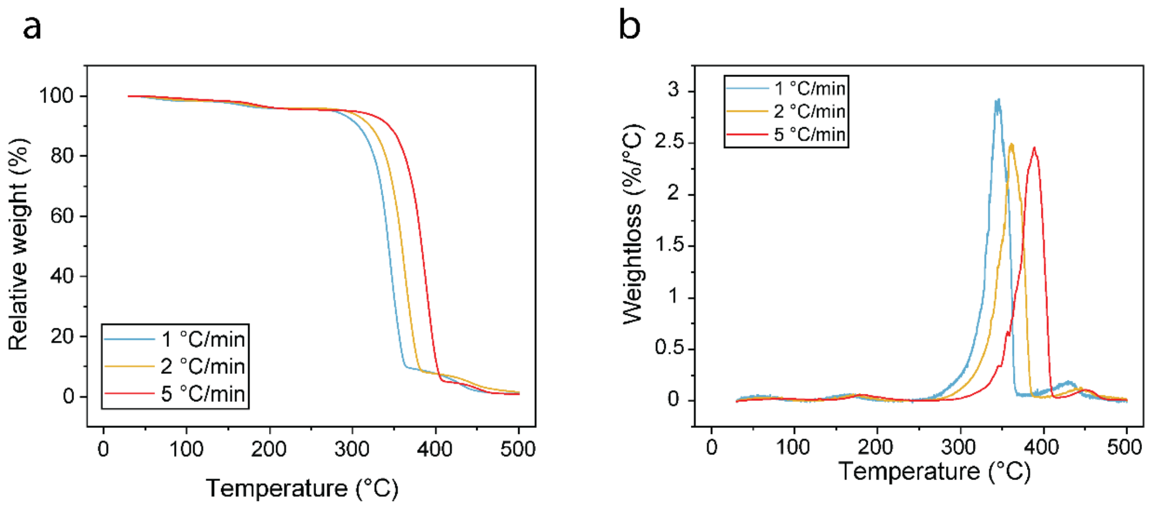

Figure S4. Pyrolysis of the polystyrene-block-poly(4-vinylpyridine) precursor at different heating rates analysed via TGA.

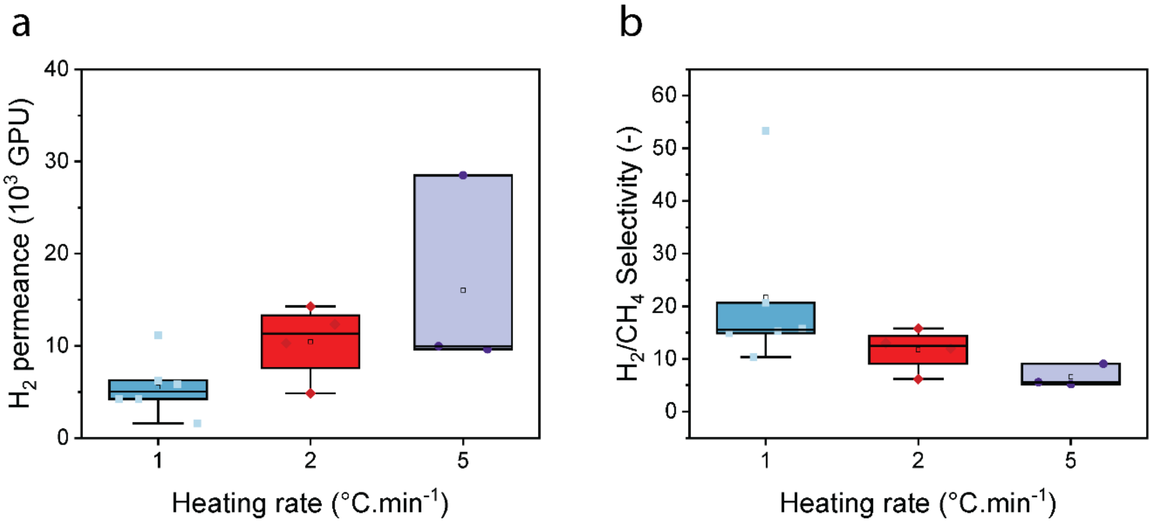

Figure S5. H_2 permeance and H_2/CH_4 separation factor for Teflon-reinforced PNG membranes prepared with different heating rates in combination with -5.3 °C sec^{-1} cooling.

1.5. Supplementary Note S5: Scaling up PNG synthesis

PNG synthesis route presented in this study is inherently simpler to upscale compared to top-down pore etching strategies. It has several advantages that are outlined below:

1. *One-step synthesis:* less complicated and less contamination risks
2. *Atmospheric pressure:* no complicated / costly vacuum equipment needed
3. *Pyrolysis process:* less dependence on atmosphere conditions compared to CVD and thus less dependant on fine control over atmosphere control

The upscaling potential of PNG was demonstrated by transitioning from a 2 cm diameter, 25 cm heating zone length tube furnace to a 13 cm diameter, 1 m heating zone length furnace. This represents about a 170x increase in furnace volume hence allowing for much larger PNG batches to be synthesized. A 4 by 7 cm^2 piece of PNG-2 was synthesized in this furnace (Figure S6). SEM imaging of the resulting PNG-2 shows the nanoporous carbon structure is obtained, as expected. To demonstrate the actual gas separation performance of the upscaled PNG, PTMSP-reinforced membranes were prepared and tested and their performance was offset against similar small-scale CVD-based membranes (Figure S7). While there naturally is some inherent variability in membrane performance, the performance of the large-scale CVD-based PNG was found not to significantly differ with CO_2 permeance ranging between 800 - 5200 GPU with $S_{CO2/N2}$ 15.7 - 22.1 .

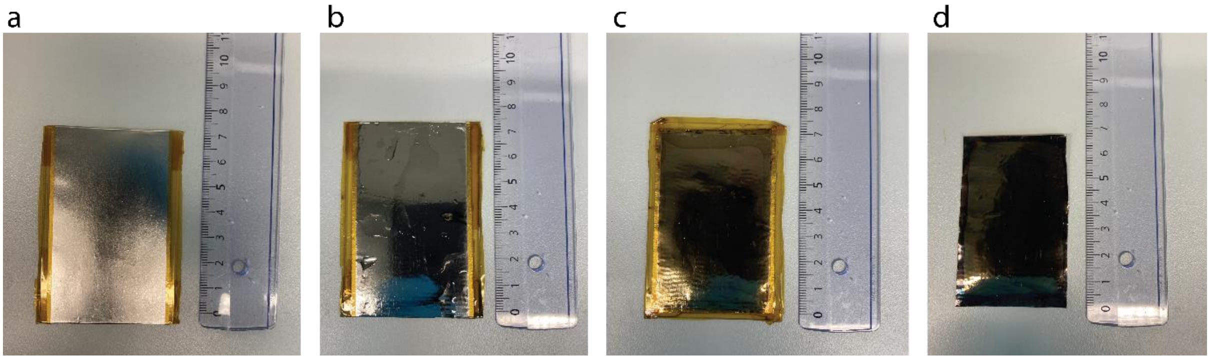

Figure S6. Images illustrating the synthesis of PNG on a 7 × 4cm^2 nickel foil showing (a) the annealed nickel foil and the foil after (b) polishing, (c) precursor coating and (d) after pyrolysis.

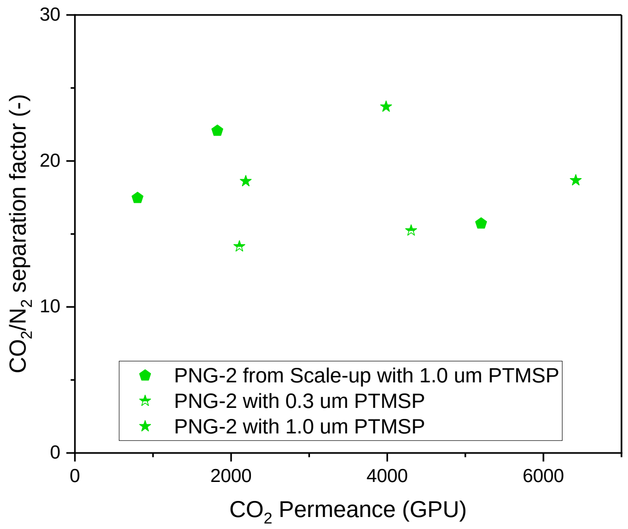

Figure S7. Comparison of the CO_2/N_2 separation performance at 35 °C of PTMSP-coated PNG prepared using the small- and large-scale CVD.

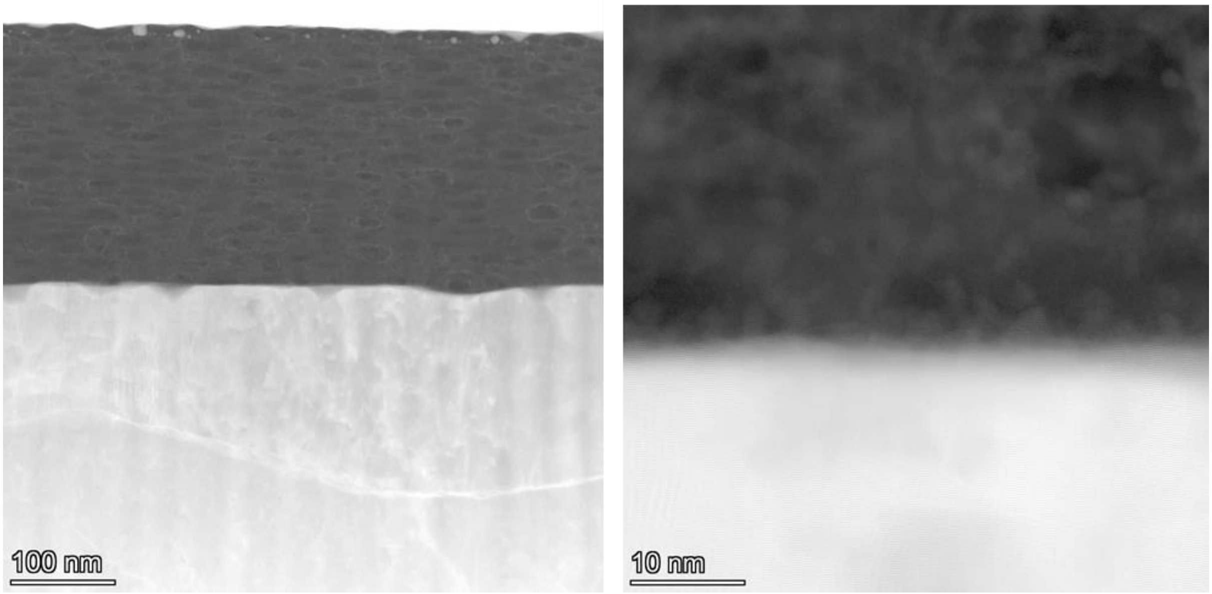

Figure S8. Low and high magnification ADF-STEM image of a PNG lamella prepared via FIB showing respectively the NPC film on the nickel foil and the graphene-nickel interface.

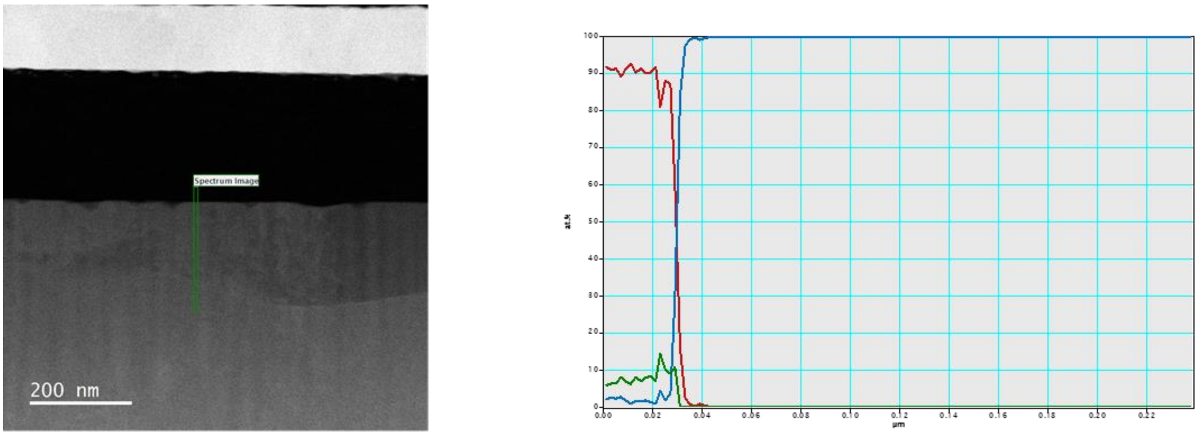

Figure S9. ADF-STEM image of a PNG lamellae prepared through FIB and the associated STEM-EELS line-scan of the area indicated by a green rectangle (blue: nickel; red: carbon; green: oxygen).

2. Detailed overview of membrane data

Table S2. Permeance data of Teflon AF-reinforced PNG-membranes synthesized with different cooling rate.

Cooling rate	Membrane	Temperature	H_2 (GPU)	CO_2 (GPU)	N_2 (GPU)	CH_4 (GPU)
-1 °C min^{-1}	M1	35 °C	3.03×10^4	2.56×10^4	3.28×10^3	4.89×10^3
-5.3 °C sec^{-1}	M1*	150 °C	2.12×10^4	8.60×10^3	1.35×10^3	1.83×10^3
		35 °C	1.12×10^4	7.49×10^3	5.77×10^2	7.31×10^2
	M2*	150 °C	3.63×10^4	1.37×10^4	3.29×10^3	4.28×10^3
		35 °C	6.25×10^3	6.86×10^3	3.91×10^2	8.90×10^2
	M3**	150 °C	9.59×10^3	3.65×10^3	6.83×10^2	1.04×10^3
		35 °C	4.21×10^3	3.03×10^3	1.92×10^2	2.67×10^2
	M4**	150 °C	1.16×10^4	3.83×10^3	6.69×10^2	8.36×10^2
		35 °C	5.83×10^3	4.17×10^3	2.89×10^2	3.91×10^2
	M5***	150 °C	8.62×10^3	3.00×10^3	4.74×10^2	5.13×10^2
		35 °C	4.21×10^3	2.69×10^3	1.42×10^2	2.04×10^2
	M6***	150 °C	3.09×10^3	6.30×10^2	8.3×10^1	8.2×10^1
		35 °C	1.60×10^3	6.25×10^2	2.7×10^1	3.0×10^1
-8.2 °C sec^{-1}	M1	150 °C	4.22×10^4	n.m.	n.m.	4.42×10^3
		35 °C	3.33×10^4	n.m.	n.m.	4.00×10^3
	M2	150 °C	4.59×10^4	n.m.	n.m.	5.21×10^3
		35 °C	3.06×10^4	1.55×10^3	2.82×10^3	3.83×10^3

Table S3. Permeance data of Teflon AF-reinforced PNG-membranes synthesized with different cooling rate.

Cooling rate	Membrane	Temperature	H_2/ CH_4	CO_2/ N_2	CO_2/ CH_4	H_2/ N_2
-1 °C min^{-1}	M1	35 °C	6.20	7.80	5.24	9.24
-5.3 °C sec^{-1}	M1*	150 °C	11.58	6.37	4.70	15.70
		35 °C	15.32	12.98	10.25	19.41
	M2*	150 °C	8.48	4.16	3.20	11.03
		35 °C	7.02	17.54	7.71	15.98
	M3**	150 °C	9.22	5.34	3.51	14.04
		35 °C	15.77	15.78	11.35	21.93
	M4**	150 °C	13.88	5.72	4.58	17.34
		35 °C	14.91	14.43	10.66	20.17
	M5***	150 °C	16.80	6.33	5.85	18.19
		35 °C	20.64	18.94	13.19	29.65
	M6***	150 °C	37.68	7.59	7.68	37.23
		35 °C	53.33	23.15	20.83	59.26
-8.2 °C sec^{-1}	M1	150 °C	9.55	n.m.	n.m.	n.m.
		35 °C	8.33	n.m.	n.m.	n.m.
	M2	150 °C	8.81	n.m.	n.m.	n.m.
		35 °C	7.99	0.55	0.40	10.85

Table S4. Permeance data of Teflon AF-reinforced PNG-membranes synthesized using increased heating rate in combination with -5.3 °C sec^{-1} cooling rate. For the data on 1 °C min^{-1} heating the reader is referred to Table S2.

Heating rate	Membrane	Temperature	H_2 (GPU)	CO_2 (GPU)	N_2 (GPU)	CH_4 (GPU)
2 °C min^{-1}	M1	150 °C	3.31×10^4	7.98×10^3	2.35×10^3	3.02×10^3
		35 °C	1.43×10^4	8.39×10^3	1.12×10^3	9.03×10^2
	M2	150 °C	1.86×10^4	7.05×10^3	1.84×10^3	2.58×10^3
		35 °C	1.23×10^4	8.38×10^3	1.13×10^3	1.98×10^3
	M3	150 °C	1.83×10^4	4.35×10^3	1.22×10^3	1.37×10^3
		35 °C	1.03×10^4	4.77×10^3	5.76×10^2	7.94×10^2
	M4	150 °C	n.m.	n.m.	n.m.	n.m.
		35 °C	4.86×10^3	3.23×10^3	2.91×10^2	4.04×10^2
5 °C min^{-1}	M1	150 °C	1.62×10^4	n.m.	n.m.	2.56×10^3
		35 °C	9.62×10^3	n.m.	n.m.	1.86×10^3
	M2	150 °C	1.74×10^4	n.m.	n.m.	2.97×10^3
		35 °C	9.96×10^3	n.m.	n.m.	1.78×10^3
	M3	150 °C	4.91×10^4	1.35×10^4	3.23×10^3	4.50×10^3
		35 °C	2.85×10^4	1.72×10^4	1.94×10^3	3.14×10^3

Table S5. Permeance data of Teflon AF-reinforced PNG-membranes synthesized using increased heating rate in combination with -5.3 °C sec^{-1} cooling rate. For the data on 1 °C min^{-1} heating the reader is referred to Table S2.

Heating rate	Membrane	Temperature	H_2/ CH_4	CO_2/ N_2	CO_2/ CH_4	H_2/ N_2
2 °C min^{-1}	M1	150 °C	10.96	3.40	2.64	14.09
		35 °C	15.84	7.49	9.29	12.77
	M2	150 °C	7.21	3.83	2.73	10.11
		35 °C	6.21	7.42	4.23	10.88
	M3	150 °C	13.36	3.57	3.18	15.00
		35 °C	12.97	8.28	6.01	17.88
	M4	150 °C	n.m.	n.m.	n.m.	n.m.
		35 °C	12.03	11.10	8.00	16.70
5 °C min^{-1}	M1	150 °C	6.33	n.m.	n.m.	n.m.
		35 °C	5.17	n.m.	n.m.	n.m.
	M2	150 °C	5.86	n.m.	n.m.	n.m.
		35 °C	5.60	n.m.	n.m.	n.m.
	M3	150 °C	10.91	4.18	3.00	15.20
		35 °C	9.08	8.87	5.48	14.69

Table S6: Table comparing the advantages and disadvantages of various pore creation strategies

Pore creation strategy	Advantages	Disadvantages
Bottom-up synthesis of pores on high-C-solubility metal (e.g., Ni) by controlled carbon precipitation (this work)	• One step synthesis of porous graphene (no additional step or equipment required for pore etching).<br>• Atmospheric pressure synthesis process.<br>• Low temperature synthesis (e.g., 500 ^oC) compared to conventional graphene synthesis (e.g., 1000 ^oC).<br>• Possibility to vary precursor, heating and cooling rate, and substrate for modulating porosity.<br>• Easy and fast process, favourable for upscaling.	• Requires high C-solubility substrate such as Ni, Pt or Rh<br>• Challenging to precisely control pore-size because pores are essentially grain-boundary defects. This work introduces rapid cooling to effectively reduce the pore size.
Bottom-up synthesis of porous graphene on low-carbon solubility metal (e.g., Cu) by defect incorporation during crystallization	• One-step synthesis<br>• Lower synthesis temperature (e.g., 900 ^oC) compared to conventional graphene synthesis (e.g., 1000 ^oC).<br>• Possibility to vary precursor, synthesis temperature, oxygen leak, etc. to change pore size and density.	• Difficult to implement this in controlled way under atmospheric pressure synthesis process.<br>• Requires complex equipment to control atmosphere conditions (pressure, composition).<br>• Challenging to decouple pore size and pore density.
Post-synthetic pore incorporation (Physical etching using ion or electron beam)	• Allows fabrication of single pore device for studying fundamental transport properties.	• 2-step procedure involving two set of equipment.<br>• Requires additional equipment for the ion/e-beam generation.<br>• Challenging to scale-up due to use of ultrahighvacuum (UHV) equipment.<br>• Challenging to control pore size in Å-regime (has not been demonstrated for gas separation) mainly because energy barrier for pore nucleation (~21 eV) is much higher than the energy barrier for pore expansion (~14 eV).
Post-synthetic pore incorporation (Chemical etching (O_2, O_2-plasma, CO_2, O_3, UV/O_3))	• Independent control over pore-size and pore density has been demonstrated for selected variants (e.g., using O_3)<br>• Pore size can be easily controlled in wide size range for gas separation to the separation of large molecules.	• 2-step procedure involving two set of equipment.<br>• Chemical usage is an additional safety risk.

References

(1) Lander, J. J.; Kern, H. E.; Beach, A. L. Solubility and Diffusion Coefficient of Carbon in Nickel: Reaction Rates of Nickel-Carbon Alloys with Barium Oxide. *J. Appl. Phys.* **1952**, *23* (12), 1305–1309. https://doi.org/10.1063/1.1702064.

(2) Yu, Q.; Lian, J.; Siriponglert, S.; Li, H.; Chen, Y. P.; Pei, S. S. Graphene Segregated on Ni Surfaces and Transferred to Insulators. *Appl. Phys. Lett.* **2008**, *93* (11), 113103. https://doi.org/10.1063/1.2982585.

(3) Li, X.; Li, H.; Lee, K. R.; Wang, A. Cooling Rate Dependence of Ni-Catalyzed Transformation of Amorphous Carbon into Graphene in Rapid Thermal Processing: An Experimental and Reactive Molecular Dynamics Study. *Appl. Surf. Sci.* **2020**, *529*, 147042. https://doi.org/10.1016/j.apsusc.2020.147042.

(4) Koh, A. T. T.; Foong, Y. M.; Chua, D. H. C. Comparison of the Mechanism of Low Defect Few-Layer Graphene Fabricated on Different Metals by Pulsed Laser Deposition. *Diam. Relat. Mater.* **2012**, *25*, 98–102. https://doi.org/10.1016/j.diamond.2012.02.014.
